# Measuring the intensity of conflicts in conservation

**DOI:** 10.1111/conl.12783

**Published:** 2021-01-11

**Authors:** Jeremy J. Cusack, Tom Bradfer‐Lawrence, Zachary Baynham‐Herd, Sofia Castelló y Tickell, Isla Duporge, Håvard Hegre, Lara Moreno Zárate, Vincent Naude, Sahil Nijhawan, John Wilson, Dario Gerardo Zambrano Cortes, Nils Bunnefeld

**Affiliations:** ^1^ Biological and Environmental Sciences University of Stirling Stirling UK; ^2^ Centro de Modelación y Monitoreo de Ecosistemas Universidad Mayor Santiago Chile; ^3^ RSPB Centre for Conservation Science 2 Lochside View, Edinburgh, EH12 9DH; ^4^ School of GeoSciences University of Edinburgh Edinburgh UK; ^5^ Interdisciplinary Centre for Conservation Science University of Oxford Oxford UK; ^6^ Wildlife Conservation Research Unit University of Oxford Oxford UK; ^7^ Peace and Conflict Research Uppsala University Uppsala Sweden; ^8^ Grupo de Gestión de Recursos Cinegéticos y Fauna Silvestre Instituto de Investigación en Recursos Cinegéticos (IREC) Ciudad Real Spain; ^9^ Institute for Communities and Wildlife in Africa University of Cape Town Cape Town South Africa; ^10^ Department of Anthropology University College London London UK; ^11^ Forest and Nature Conservation Policy Group Wageningen University Wageningen The Netherlands

**Keywords:** armed conflict, coexistence, curve, deescalation, discourse, escalation, spatiotemporal, stakeholder groups, stasis, trend

## Abstract

Conflicts between the interests of biodiversity conservation and other human activities pose a major threat to natural ecosystems and human well‐being, yet few methods exist to quantify their intensity and model their dynamics. We develop a categorization of conflict intensity based on the curve of conflict, a model originally used to track the escalation and deescalation of armed conflicts. Our categorization assigns six intensity levels reflecting the discourse and actions of stakeholders involved in a given conflict, from coexistence or collaboration to physical violence. Using a range of case studies, we demonstrate the value of our approach in quantifying conflict trends, estimating transition probabilities between conflict stages, and modeling conflict intensity as a function of relevant covariates. By taking an evidence‐based approach to quantifying stakeholder behavior, the proposed framework allows for a better understanding of the drivers of conservation conflict development across a diverse range of socioecological scenarios.

## 1 INTRODUCTION

Efforts to conserve biodiversity are often at odds with the needs and interests of other human activities—such as agriculture (Shackelford, Steward, German, Sait, & Benton, 2015) or urban development (Moilanen et al., 2011)—leading to widespread conservation conflicts. Common examples include conflicts surrounding the management of threatened species that impact human livelihoods and food security (Cusack et al., 2019; Van Eeden et al., 2018), the establishment of protected areas that displace local people (Soliku & Shraml, 2018), or the regulation of harvesting activities to ensure sustainable use of natural resources (Cusack et al., 2020; Yasmi, Schanz, & Salim, 2006). Redpath et al. (2013) define such conflicts as “situations that occur when two or more parties with strongly held opinions clash over conservation objectives and when one party is perceived to assert its interests at the expense of another.” Yet, despite the potential for conservation conflicts to negatively impact both biodiversity conservation and human well‐being, there currently exists no standardized approach for measuring their intensity.

Attempts to quantify conservation conflicts have so far given considerable importance to the actual impacts or costs—be they ecological, economic or societal—that different stakeholders experience as a result of conservation actions (Redpath et al., 2013; Young et al., 2010). A common example of this is livestock loss as a result of predation by protected large carnivores, and the ensuing retaliatory killing of carnivore species by affected people (Van Eeden et al., 2018). Importantly, such measures implicitly frame the conflict as occurring between humans and wildlife (so‐called human–wildlife conflict; Redpath, Bhatia, & Young, 2015), when in reality they are indicators of a larger conservation conflict characterized by the attitude and behavior of different human interest groups towards one another (Colvin, Witt, & Lacey, 2015; Dickman, 2010; Madden & McQuinn, 2014; Redpath et al., 2015; Zimmermann, McQuinn, & Macdonald, 2020). These human–human interactions, which may be shaped by both long and short‐term histories, cannot easily be captured by proxy measures of loss or gain.

More recent efforts to measure conservation conflict intensity have sought to quantify incompatibilities between the interests of conservation and other human activities by investigating patterns of spatial overlap (Kehoe et al., 2015; Shackelford et al., 2015) or quantifying consensus towards a given topic (e.g., the potential for conflict index; Vaske, 2018). Although valuable, approaches such as these overlook the actions that different interest groups perform in response to one another, which can range from cooperative to antagonistic (Madden & McQuinn, 2014; Zimmermann et al., 2020). Most importantly, existing measures of conservation conflict intensity are case‐specific and thus challenging to generalize across species, conservation issues, or geographic areas (Inskip & Zimmermann, 2009; Soliku & Schraml, 2018). This has hindered comparative studies of conservation conflict development and prevented broader‐scale synthesis of the drivers causing conflict escalation or deescalation.

In this study, we develop a categorization of conservation conflict intensity based on the curve of conflict model used to describe the escalation and deescalation of armed conflict (Crowley, Hinchliffe, & McDonald, 2017; Lund, 1996). Research into the occurrence and characteristics of armed conflicts at a global scale has greatly benefitted from categorizations of conflict type and intensity (Gleditsch, Wallensteen, Eriksson, Sollenberg, & Strand, 2002; Trinn & Wencker, 2018). These have enabled a better understanding of the factors driving the initiation and maintenance of armed conflict (Diehl, Goertz, & Gallegos, 2019), but also paved the way for the development of predictive models of conflict escalation and deescalation (Hegre, Karlsen, Nygård, Strand, & Urdal, 2013; 2019). Despite differences in the levels of violence involved, the development of conservation and armed conflicts share similarities. First, both involve the imposition of one or several interests over those of others, resulting in situations of dominance or discord. Second, both are characterized by a combination of political discourse and concrete actions that can be used to infer conflict intensity. Third, both armed conflicts and conservation conflicts typically involve a number of parties, each of whose actions can cause the conflict to escalate or deescalate over time. Lastly, like conservation conflicts, armed conflicts can vary in their historical and geopolitical contexts. We make use of these similarities to identify six levels of conservation conflict. We first outline general characteristics for each level that allow for standardized categorization as well as spatiotemporal flexibility. We then demonstrate the value of our approach using a range of case studies, highlighting common patterns and drivers of conflict escalation and deescalation.

## 2 THE CURVE OF CONFLICT

**FIGURE 1:**
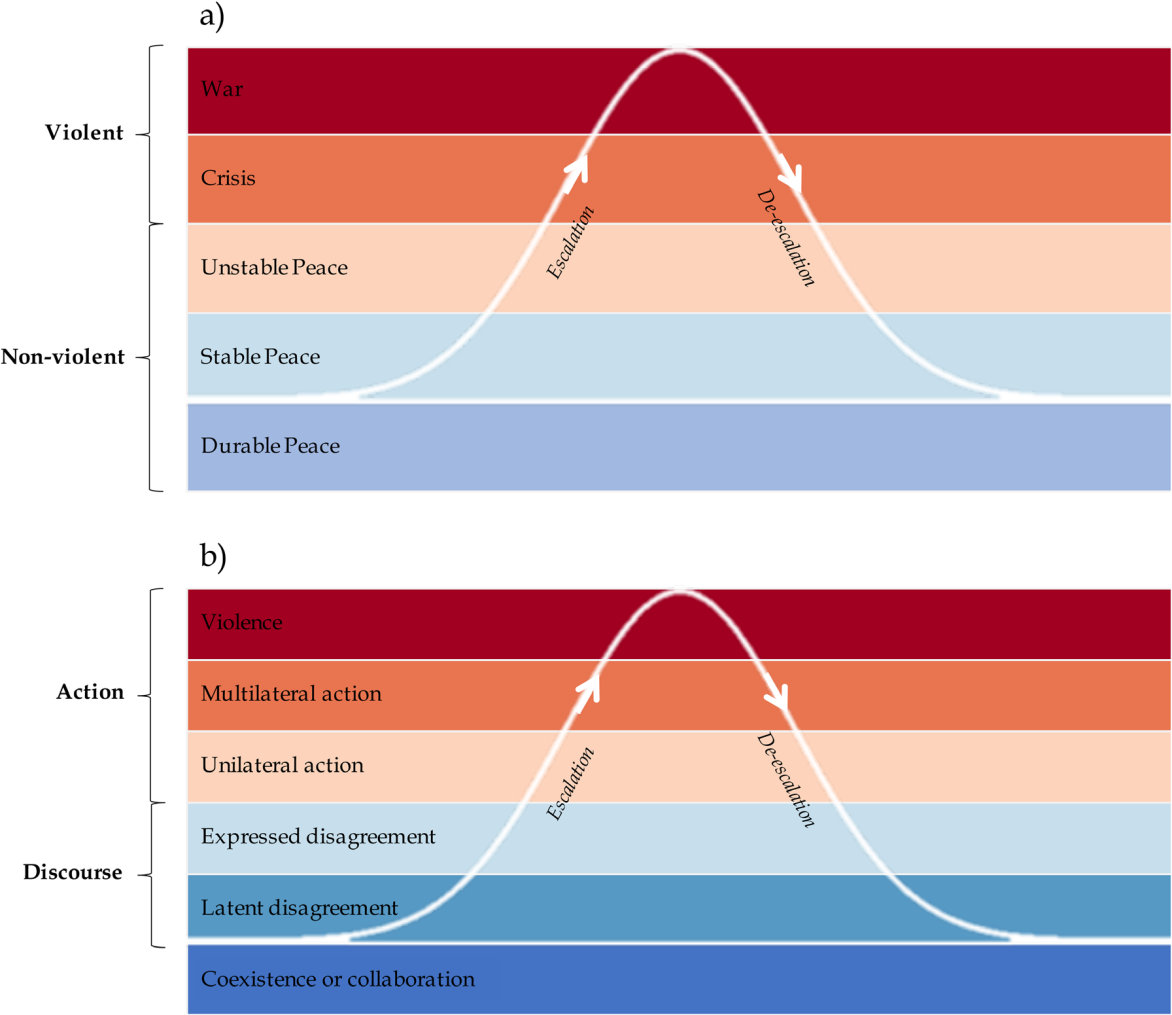
Schematic representation of (a) Lund's (1996) curve of conflict and (b) the proposed conservation conflict curve

The curve of conflict is a conceptual model that illustrates the rise and fall of conflict intensity over time (Lund, 1996; Figure 1a). It was developed with the aim of guiding armed conflict prevention and shows how different conflict phases relate to one another and to various kinds of third‐party interventions. The curve also helps to organize terms and concepts used by conflict management professionals. In particular, Lund (1996) separates conflict into nonviolent (*Durable*, *Stable* and *Unstable Peace*) and violent (*Crisis* and *War*) stages, highlighting conflict management interventions that relate to these different levels of intensity.

The curve of conflict describes a state of *Durable Peace* as being characterized by a high level of reciprocity and cooperation, including shared values, goals and institutions (Lund, 1996). Transition to a state of *Stable Peace* occurs when cooperation and communication between parties becomes wary, for example, as a result of differences in goals and values. In contrast, *Unstable Peace* is characterized by increased tension and suspicion, with positions becoming increasingly polarized. Armed forces may be used as a deterrent. Higher levels of conflict involve open violence, first as open mobilization of armed forces and low‐level skirmishes (*Crisis*), then as sustained violent fighting that may engender a spiral of escalating violence (*War*). When actors cross the threshold of nondeniable overt violence, dynamics typically change fundamentally—past violence is by far the best predictor of continued violence (Hegre, Hultman, & Nygård, 2019).

## 3 THE CONSERVATION CONFLICT CURVE

The intuitive and broadly applicable trajectory described by the curve of armed conflict enables us to adapt this conceptual model to the case of conservation conflicts (Figure 1b). The resulting conservation conflict curve identifies six conflict intensity levels (Table 1): coexistence or collaboration (level 0); latent disagreement (level 1); expressed disagreement (level 2); unilateral action (level 3); multilateral action (level 4); and physical violence (level 5). Conflict initiation occurs as a result of a trigger, defined as a discourse or action causing the emergence of conflicting interests surrounding a conservation issue. Here, the term stakeholder refers to any group with a clearly defined interest in the topic causing the conservation conflict. Importantly, identification of stakeholders should be based on stated interests rather than thematic groups. For example, two conservation NGOs with contrasting views on the value of trophy hunting to protect a given species should be considered as separate stakeholders. Although we acknowledge that individuals within a stakeholder group often hold personal views, these are not considered further in the present study.

**TABLE 1:** Definition and characterization of conservation conflict intensity levels

Level	Definition	Characterization	References
0. Coexistence or collaboration	Interests of conservation and other human activities do not compete but work alongside each other.	● Lack of negative discourse and actions reflecting opposing interests, both within and amongst stakeholder groups. ● Evidence for collaborative actions or discourse.	Butler et al. (2015); Raithel, Reynolds‐Hogland, Koons, Carr, and Aubry, (2017); Cusack et al. (2019)
1. Latent disagreement	An underlying conflict that is not apparent or visible in the interaction between different stakeholders.	● Negative discourse held among members of a given stakeholder group about the interests of other stakeholder groups. ● May follow from a situation of coexistence that has begun to break down. ● May occur when conflict symptoms have been resolved but underlying causes have not.	Madden and McQuinn (2014)
2. Expressed disagreement	Conflict is visible in the discourse and dialogue exchanged between different stakeholder groups, but no concrete actions are undertaken to influence interests.	● Disagreements recorded in both written and spoken forms, such as within traditional and social media outlets, or during face‐to‐face meetings.	Hodgson, Redpath, Fischer, and Young (2018)
3. Unilateral action	A single stakeholder group carries out one or more activities related to the conservation issue at hand that directly influence the interests of other stakeholders.	● Actions surpass discourse but do not involve physical violence. ● Examples might include the enactment of policy or law, illegal activities, boycotting or lobbying, peaceful demonstrations, the gazetting of a protected area against the wishes of local stakeholders, the listing of a species on CITES, wildlife management of any kind, or voluntary inaction. ● Unilateral actions may be prominent in conflicts involving significant power imbalances.	Redpath et al. (2013); Aiyadurai (2016); Cusack et al. (2020)
4. Multilateral action	More than one stakeholder group carries out one or more actions related to the conservation issue at hand.	● Actions are antagonistic, that is, they seek to defend each group's interests. ● More groups have been sufficiently motivated to take action, and therefore conflict intensity has increased compared to when only a single group has taken action. ● Collaborative actions do not contribute towards this level (see level 0).	Spijkers et al. (2018); Cusack et al. (2020)
5. Physical violence	Conflict characterized by extreme actions carried out by stakeholder groups that cause human injury or death.	● Examples include the involvement of armed forces in preventing illegal activities, riots, enforced land clearances, or the murder of activists.	Barbora (2017)

An important aspect of the conservation conflict curve is its spatiotemporal flexibility. It can be applied to any given spatial unit that is deemed to most adequately represent the scale at which the focal conflict occurs. Conflicts can be very local, such as a disagreement between two individuals over the management of common land harboring biodiversity, or international in the case of global bans on valuable wildlife products affecting relations between two or more countries (Dickman, Cooney, Johnson, Louis, & Roe, 2019). Importantly, stakeholder discourse and actions should refer to disagreements that relate to conservation measures carried out within the chosen spatial unit.

Conflict level is also assessed over a user‐defined time step. Repeated assessments over a series of time steps provide a trend in conflict intensity (see case studies below). Time step length can be chosen to reflect the dynamic nature of stakeholder interactions, which may change over daily to decadal timescales. Importantly, choice of both the spatial unit and time step length will be influenced by the resolution of the data available for assessing intensity (Hegre et al., 2019). We recommend selecting a spatiotemporal unit that minimizes gaps in the measurement of intensity over time.

## 4 CONFLICT ESCALATION, DEESCALATION, AND STASIS

**FIGURE 2:**
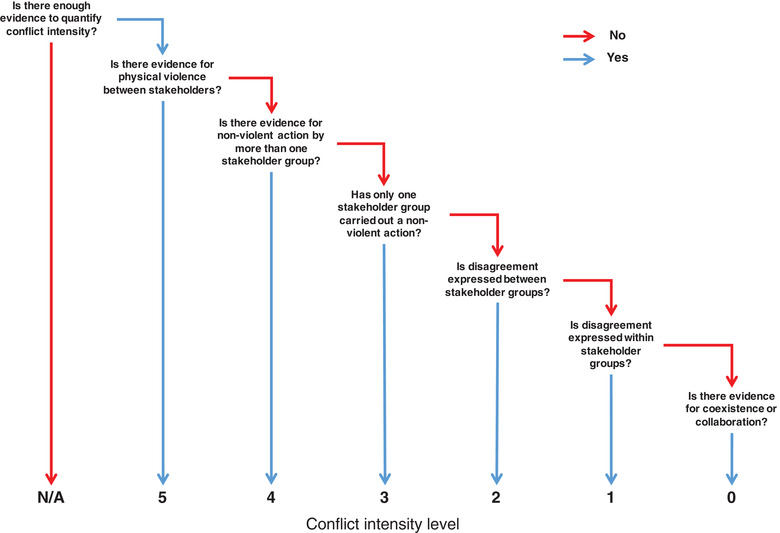
Decision tree to assign intensity levels using the conservation conflict curve. The term “action” relates to any activity carried out by a stakeholder that directly influences the interests of other stakeholder groups

Conflict intensity during a single time interval is assumed to be the most severe level for which there is reliable evidence (Figure 2; Lund, 1996). If the evidence suggests a higher intensity level than the previous time step, conflict intensity has escalated. Conflict level is reassessed at each time step, considering the potential temporal dependency on the preceding year. The latter occurs when events at time *t* – 1 become the status quo at time *t*. For example, introduction of new legislation or management policy by a stakeholder would indicate a conflict is at level 3 (unilateral action, assuming no other actions or violence are occurring). By the following time step, the legislation or management policy has become embedded and represents the new status quo, i.e. it does not represent an action undertaken at time *t*. If no new action has been performed during time step *t*, a lower level of conflict intensity would be assigned based on available evidence (i.e., deescalation). Importantly, actions representing collaborative efforts by stakeholders to address the issue causing a conflict should be assigned level 0. In other words, they do not contribute towards multilateral action (level 4), even though this level can still occur during the same time step if collaborative actions by multiple stakeholders occur alongside noncollaborative actions.

Reassessment of conflict intensity at each time step can also reveal periods of stasis. Many armed conflicts, for example, involve periods of stasis interspersed by phases of rapid shift (Diehl & Goertz, 2001). Stasis may take place at any level of conflict intensity and occurs when there is repeated evidence of a particular conflict level over successive time intervals. For example, if repeated assessment of a conflict reveals evidence of illegal harvesting of a protected species every year, then the conflict will be at a constant level 3 (again, assuming no other actions are occurring other than illegal harvesting).

## 5 EVIDENCE FOR STAKEHOLDER DISCOURSE AND ACTIONS

The proposed categorization of conservation conflict intensity relies on the synthesis of evidence reflecting the discourse and actions of all relevant stakeholders (Figure 2). Evidence may be assembled from a variety of published sources, such as peer‐reviewed scientific articles, gray literature (e.g., report from governmental or nongovernmental organizations), stakeholder websites, meeting minutes or traditional media outlets, in addition to social media channels (Killion, Melvin, Lindquist, & Carter, 2019). Conflict intensity may also be derived directly from social surveys, such as questionnaires and online surveys targeted at specific stakeholders (Ainsworth, Redpath, Wilson, Wernham, & Young, 2020). Anecdotal evidence may also be used to corroborate existing evidence or provide additional context to the conflict situation. The resulting collection of discourses and actions can be used to elaborate a timeline of conflict development, which can subsequently be divided according to the chosen time step.

## 6 APPLICATION TO CONSERVATION CONFLICT CASE STUDIES

**FIGURE 3:**
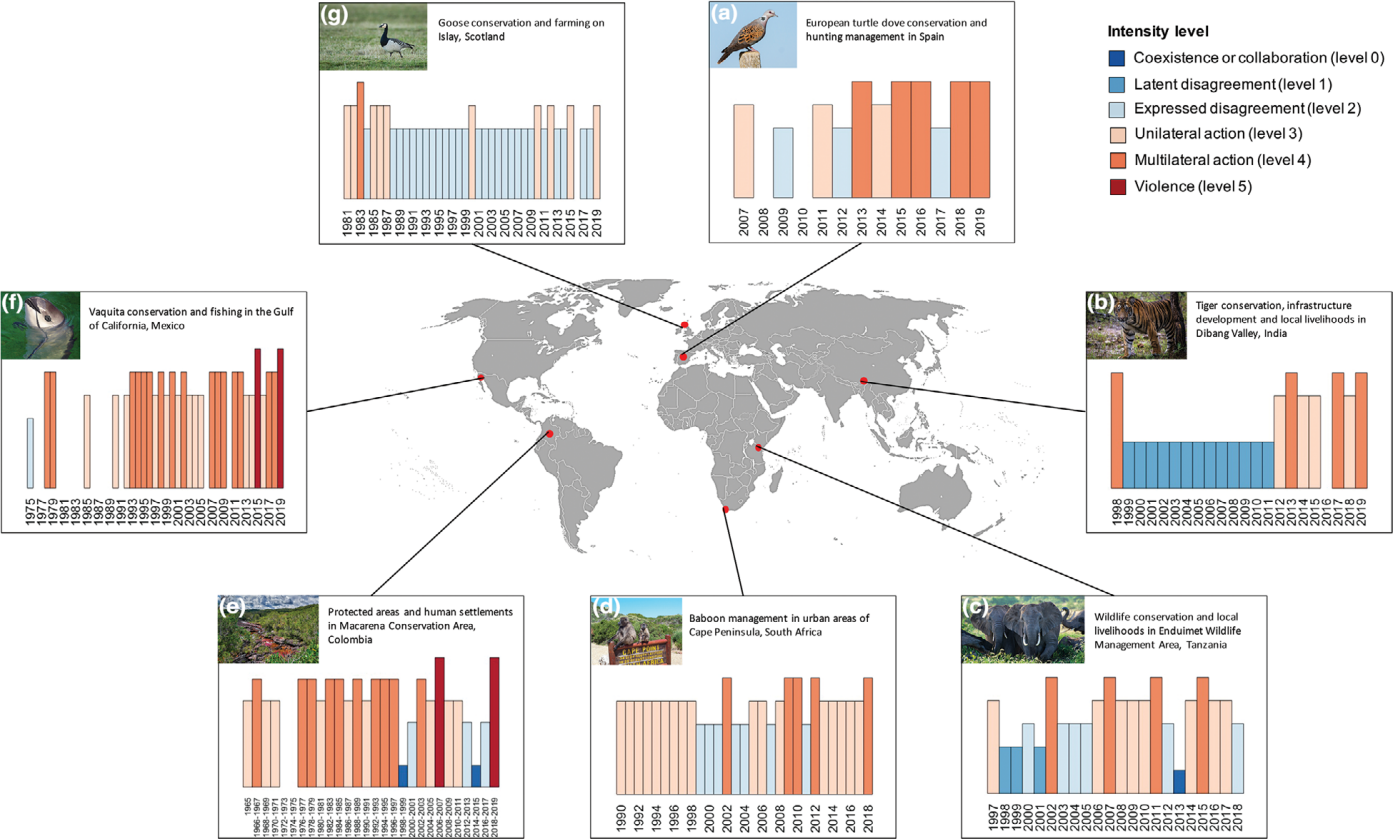
Time series of conflict intensity level for the different case studies (a–g). Each conflict characterization begins with a trigger and is measured for a given time step length (see Table 2). Missing bars denote absence of evidence for a given time step

As a proof of concept, we applied the conservation conflict curve to seven conservation conflict case studies (Table 2; Figure 3; Supporting Information S1). Case studies were assembled by participants of the Interdisciplinary Conservation Network workshop held in Oxford, UK, in July 2018. Each participant was asked to identify a conflict they were familiar with prior to the workshop. Following the workshop, participants were asked to determine a conflict trigger and assign intensity levels for their case study based on a time interval that best reflected available evidence (Supporting Information S2). Assigned conflict levels were subsequently re‐assessed by either the first or second author to provide an objective evaluation. Comparison of first and second assessments provided an average matching of 92% across case studies (range: 84–100%). Taken together, the selected case studies consist of a diverse and representative sample of existing conservation conflicts, which we use here to provide a proof of concept for the proposed approach (Supporting Information S3).

**TABLE 2:** Summary of case study time series

Case study	Trigger (year)	Time span	Time step length	# Time steps (with evidence)
European turtle dove conservation and hunting management in Spain	Species Action Plan (2007)	2007–2019	1 year	13 (11)
Tiger conservation, infrastructure development and local livelihoods in Dibang Valley, India	Establishment of Dibang Valley Wildlife Sanctuary (1998)	1998–2019	1 year	22 (21)
Wildlife conservation and local livelihoods in Enduimet Wildlife Management Area, Tanzania	Proposal to establish Enduimet Wildlife Management Area (1997)	1997–2018	1 year	22 (22)
Baboon management in urban areas of Cape Peninsula, South Africa	Culling of entire baboon troop on Cape Peninsula (1990)	1990–2018	1 year	29 (29)
Protected areas and human settlements in Macarena Conservation Area, Colombia	Establishment of Macarena Natural Reserve (1965)	1965–2019	2 years	29 (26)
Vaquita conservation and fishing in the Gulf of California, Mexico	International Whaling Commission concerns about vaquita mortality in totoaba fisheries (1975)	1975–2019	1 year	45 (31)
Goose conservation and farming on Islay, Scotland	Wildlife and Coutryside Act (1981)	1981–2019	1 year	40 (38)

To further illustrate the value of our approach as a basis for understanding common patterns and drivers of conservation conflict escalation and deescalation, we quantified conflict transition probabilities and compared mean stasis length for the different intensity levels. Transition probabilities were derived by dividing the total number of instances of each possible transition (with six levels, *N* = 36) by the total number of transitions observed in the conflict intensity time series. Mean stasis length was calculated based on all case studies with time step length equal to 1 year, thus excluding conflict in the Macarena Conservation Area for which time step length was set to 2 years (Table 2).

We also modeled conflict level as a function of relevant covariates using a mixed effects ordinal regression approach. The response was an ordinal variable representing conflict intensity measured at time *t*. As examples of relevant covariates, we included the proportion of collaborative actions carried out a *t* – 1 (number of actions involving collaboration between multiple stakeholders divided by the total number of actions at *t* – 1; see Supporting Information S2) and the number of chronological years since the conflict trigger. We compared four model formulations (additive effect of collaboration and years since trigger; collaboration only; years since trigger only; no covariates) based on Akaike's Information Criteria (AIC) and considered the effects of all variables contained in models within 2 delta AIC of the top model. All models included case study as a random intercept and were implemented via the clmm function in the R package *ordinal* (Christensen, 2019). For the purpose of this analysis, we excluded levels 0 and 5 owing to small sample sizes and did not consider data from the Macarena Conservation Area case study to maintain a consistent 1‐year time step (Table 2).

## 7 PATTERNS AND DRIVERS OF CONFLICT INTENSITY

**FIGURE 4:**
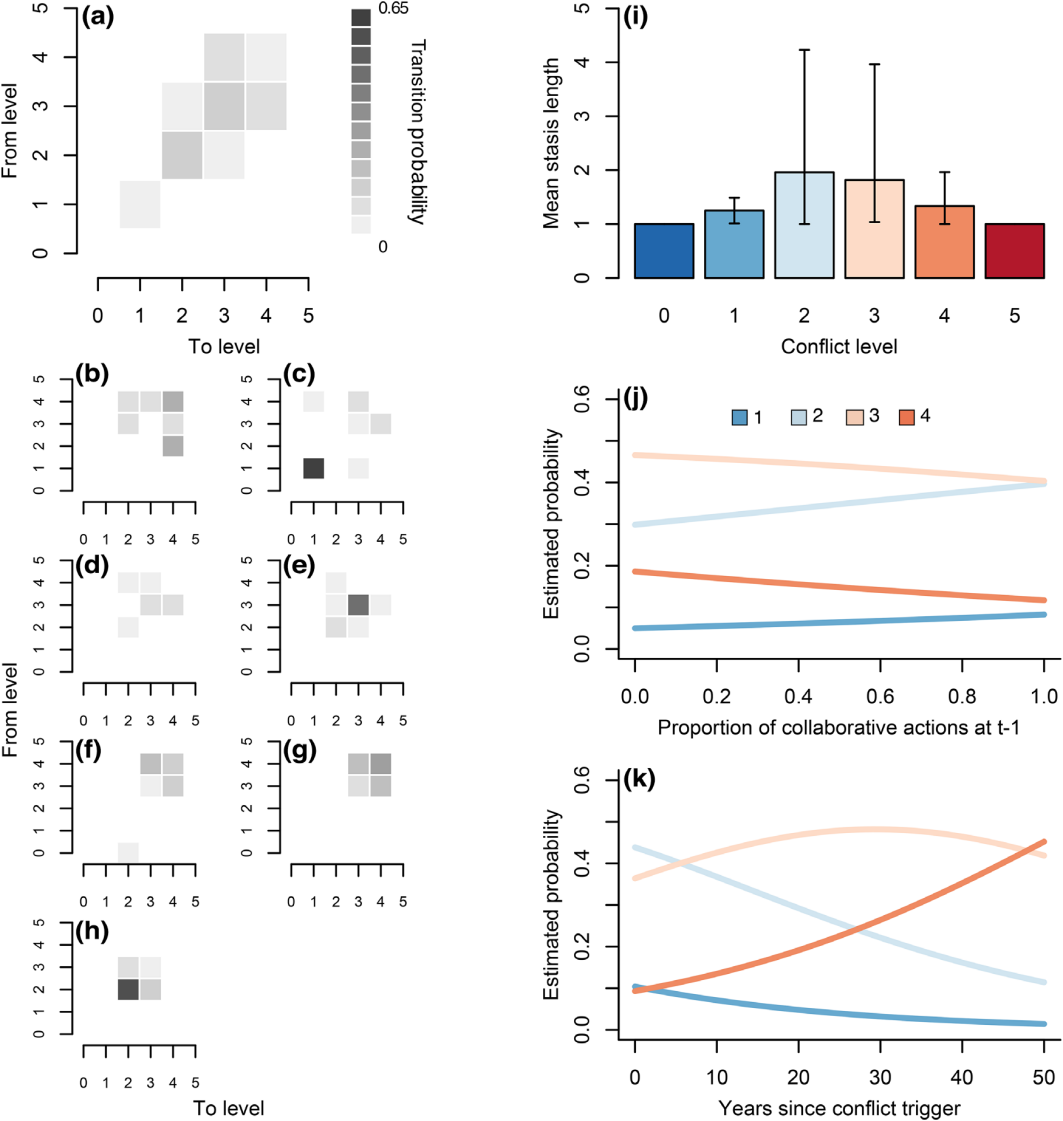
Patterns and drivers of conservation conflict escalation and deescalation, including level transition probability matrices for pooled (a) and individual (b‐h) case studies, mean stasis length for the different conflict levels (i), and influence of proportion of collaborative actions at time *t –* 1 (j) and number of years since the conflict trigger (k) on the probability of observing conflict levels 1–4. Case studies considered were: European turtle dove conservation and hunting management in Spain (b). Tiger conservation, infrastructure development and local livelihoods in Dibang Valley, India (c). Wildlife conservation and local livelihoods in Enduimet Wildlife Management Area, Tanzania (d). Baboon management in urban areas of Cape Peninsula, South Africa (e). Protected areas and human settlements in Macarena Conservation Area, Colombia (f). Vaquita conservation and fishing in the Gulf of California, Mexico (g). Goose conservation and farming on Islay, Scotland (h). Error bars in (i) denote 95% confidence intervals associated with the mean across case studies. Relationships in (j) and (k) were obtained from a mixed effects ordinal regression model that included both variables as additive fixed effects and case study as a random intercept. For clarity, relationships are shown without confidence intervals, but these can be visualized in Supporting Information S4

Conflict transition probability matrices for both pooled and individual case studies indicated a tendency for conflict time series to consist of only a small subset of all possible level transitions (Figures 4a–h). In particular, transitions between levels 2, 3, and 4 were often associated with higher probabilities relative to other transitions, most likely due to their more frequent occurrence in conflict time series (Figure 3). A notable exception was found in the case of the conflict involving tiger conservation in Dibang Valley, for which stasis at level 1 (latent conflict) was most prominent (Aiyadurai, 2016; Supporting Information S3). Mean stasis length based on a time step length of 1 year was highest and most variable across case studies for levels of conflict intensity 2 (1.96, 95% CI [1–4.23]) and 3 (1.82 [1–3.97]) (Figure 4i), while other conflict levels exhibited shorter periods of stasis on average.

Both the proportion of collaborative actions at *t* – 1 and the number of years since the conflict trigger were retained as predictors in the top ordinal regression model, highlighting their influence in determining the occurrence of conflict levels 1–4. In particular, the proportion of collaborative actions at *t* – 1 was positively associated with an increased probability of observing level 1 or 2 at time *t*, whilst it decreased the probability of observing level 3 or 4 (Figure 4j; see Supporting Information S4 for results with confidence intervals). These results highlight the positive influence of collaboration in driving conflict deescalation. In contrast, the number of years since the conflict trigger had a positive effect on the probability of observing level 4 and a negative effect on the probability of observing level 1 or 2 (Figure 4k; Supporting Information S4). The relationship was notably nonlinear in the case of level 3, with higher probabilities between 20 and 40 years into a conflict relative to earlier or later periods. Lastly, there was significant variation in the random intercept associated with individual case studies (likelihood ratio test comparing models with and without the random intercept: *χ* ^2^ = 25.9, df = 3, *p* < .001), highlighting the importance of accounting for interconflict differences.

## 8 DISCUSSION

The approach presented here provides a standardized method for quantifying the intensity of conservation conflicts based on the nature of interactions between relevant stakeholders. It is grounded in conceptual advances originating from the study of armed conflicts, and thus represents a novel interdisciplinary tool for the study of what are most often social conflicts centered around conservation issues (Redpath et al., 2013). In particular, we document clear phases of conflict escalation, stasis and temporary deescalation across a range of case studies (Supporting Information S3), thus highlighting the value of our classification in comparing the development of complex conflict situations occurring in different socioecological contexts. Moreover, we illustrate how our approach results in a measure of conflict intensity that can be used to model the drivers of conflict escalation and deescalation. In particular, our approach confirms that collaboration is key to achieving lower levels of conflict (Young et al., 2016a), while conversely, the longer a conflict lasts the more likely it is to involve stakeholder actions. Our work thus validates a proof of concept for modeling intensity as a function of a range of socioecological attributes, both within and across conflict case studies, with the aim of guiding conflict resolution strategies (Young et al., 2016b).

It is notable that none of the seven case studies presented in this work demonstrated a long‐lasting deescalation of conflict to coexistence or collaboration. On the contrary, conflicts have tended to stabilize at higher levels of intensity in recent years. Cases of coexistence do exist, as exemplified by the adaptive comanagement strategy successfully implemented to resolve the conflict between seal conservation and salmon fisheries in Scotland (Butler et al., 2015). However, in many cases there is a tendency for conflict escalation rather than resolution. This reinforces the urgent need to research and promote workable solutions based on a nuanced understanding of the social processes underlying conservation conflicts (Baynham‐Herd, Redpath, Bunnefeld, Molony, & Keane, 2018; Colvin et al., 2015; Crowley et al., 2017). In this context, the curve of conservation conflict provides a useful complement to existing conceptual frameworks (Young et al., 2016b; Zimmermann et al., 2020), supporting both a detailed qualitative analysis of individual case studies and a more quantitative investigation of general conflict trends.

It is important to acknowledge the limitations of our approach. First, absence of evidence should not be equated to lack of conflict, and we recommend accompanying assessments with detailed reference to supporting material. In so doing, it is also important to consider inherent biases that might occur in available evidence, especially in conflicts involving significant power imbalances. Such biases might occur in media articles and reports produced by one stakeholder, and even in scientific publications that push a certain research agenda or are influenced by the disciplinary background of authors (Baynham‐Herd, Redpath, Bunnefeld, & Keane, 2020). When collating evidence supporting intensity levels, we recommend corroborating discourses and events occurring at each time step using as wide a variety of sources as possible. We also recommend obtaining intensity evaluations from multiple assessors in order to minimize inherent biases associated with prior experience of a given conflict, as was implemented in this study. Second, although assignment of intensity levels can be adapted to a range of spatial and temporal resolutions, we caution against comparing conflict trends derived using two different time step lengths. Increasing time step length leads to an amalgamation of available evidence, and thus a higher likelihood of stronger conflict intensity.

In summary, we present a flexible framework for assessing the intensity of conservation conflicts. Such a tool is crucial at a time of increasing expansion of human activities into wild areas and concomitant intensification of anthropogenic land use (Díaz et al., 2019). Using a range of representative case studies from around the world, we demonstrate how our framework can be used to track the development of conservation conflicts over time, thereby opening up avenues for predictive approaches to addressing threats to both biodiversity conservation and human well‐being (Nicholson et al., 2019).

## AUTHOR CONTRIBUTIONS

JJC, TB‐L, JW, HH, and NB conceived and developed the study; JJC and TB‐L collected the data, carried out the analysis, and wrote the manuscript; ZB‐H, SCyT, ID, LM‐Z, SN, and DGZC contributed and analyzed case studies. All authors contributed toward manuscript revisions.

## ETHICS STATEMENT

Not applicable.

## DATA ACCESSIBILITY STATEMENT

All data used in this study are available in the Supporting Information.

## CONFLICT OF INTEREST

The authors declare no conflict of interest.

## Supporting information

Supplementary Material

TABLE S2 Evidence for conflict levels for each of the case studies

Supplementary Material

Supplementary Material
