# Supplementary material for: Measuring the intensity of conflicts in conservation
Source: Conserv Lett. 2021 Jan 11;14(3):e12783. doi: 10.1111/conl.12783 (PMC8365684; doi:10.1111/conl.12783)
Supplement: Supplementary file 1 — Supplementary Material [file CONL-14-e12783-s003.docx]

**Supporting Information S1:** Case study backgrounds.

a) European turtle dove conservation and hunting management in Spain.

The European turtle dove (*Streptopelia turtur*) is a migratory species whose population has declined by 78% in Europe since the 1980s (PECBMS, 2015). This decline has been particularly marked in western Europe (e.g. 94% in the UK and 37% in Spain between 1996-2018; Moreno-Zarate et al., 2020). The turtle dove is considered a quarry (i.e. game) species in 10 EU countries, with Spain having the highest yearly harvests (around 800 000 birds) (Lormée et al., 2019). Concern about the status of the turtle dove reached the public arena as a debate on environmental NGO and hunter association websites in 2012-2013. Environmentalists point to hunting as one of the main drivers of turtle dove decline, promoting a hunting moratorium for the species or the classification of turtle dove as a protected species, which would imply their exclusion from the quarry species list. Hunter associations defend the importance of their actions as key habitat management for the species and reject the idea of a moratorium. They defend the legality and sustainability of hunting. The conflict has largely remained at level 4 since the development of a species action plan in 2016 (where a hunting moratorium is contemplated specifically). In 2019, the European Commission issued a statement on a possible sanction of the Spanish government due to non-compliance of the species action plan that has intensified the differences between conservation and hunting interests.

b) Tiger conservation, infrastructure development and local livelihoods in Dibang Valley, India

Dibang Valley lies in the Northeast Indian border state of Arunachal Pradesh and is the ancestral homeland of the animistic Idu Mishmi people. The Indian Constitution limits non-native people from seeking entry or residency into the state and has granted *de facto* tenure and management rights over land and forests to local communities as per their traditional customary arrangements. Idus believe in kinship with the tiger and do not kill the species. They also observe strict ritual restrictions on the hunting of all large animals, the cutting of trees, and overfishing (Nijhawan and Mihu, 2020). Consequently, a unique population of mountain tigers has thrived in Dibang away from conservation attention and without any state or NGO-led protection mechanisms (Nijhawan, 2018). The establishment of Dibang Wildlife Sanctuary in 1998, although devoid of any enforcement on the ground, resulted in a state of latent conflict characterised by local unease (Aiyadurai, 2016). This situation changed in 2012 when state forest agencies and NGOs rescued two orphaned tiger cubs from a dry well. Dibang’s tigers have since attracted increasing attention from researchers and state authorities, with proposals to upgrade Dibang Wildlife Sanctuary to a strict government-managed tiger reserve (Aiyadurai, 2016). Recent plans by the Indian government to develop 17 mega-hydropower dams in Dibang Valley have strongly contributed to the growing conflict between local communities and advocates of tiger conservation. Under persistent pressure from the State and dam proponents, and after years of resistance, the Idu eventually agreed to the development of dams. With promises of large monetary compensation, some Idu have over time become strong supporters of the hydro-projects. In 2017, based on results of scientific research, a statutory committee of the Government of India put the construction of the largest hydropower project in Dibang Valley on hold until a systematic study of its impacts on tigers was conducted. The Idu do not understand why a reserve is needed for the tigers that they argue their culture has protected and see it as a threat to their access to forest resources and future development. The tiger, with which the Idu have co-habited for millennia, is now being seen as a hindrance to both development and aspirations for economic prosperity. In 2019, the Idu banned all future research on Dibang’s wildlife until the government clarifies its plans for the tiger reserve and dam project, thus escalating this newly created conservation conflict between local communities, State and private developers, and advocates of tiger conservation to level 4.

c) Wildlife management and local livelihoods in Enduimet Community Wildlife Management Area, Tanzania

Enduimet Community Wildlife Management Area (ECWMA) is a wildlife corridor in the Amboseli-Kilimanjaro ecosystem, northern Tanzania, where conflicts between local communities and elephant (*Loxodonta africana*) conservation are rife and damaging (Homewood, 2017). Since its proposal in 1997 and subsequent creation in 2007, ECWMA has been hotly contested locally. Local communities lament the associated resource-use restrictions, centralisation of tourism income, and increases in wildlife-related damage, including from elephants (Benjaminsen et al. 2013). Like elsewhere in the east African rangelands (Evans and Adams, 2016), elephants in ECWMA frequently raid crops and occasionally injure people. Elephants are protected by national-level regulations and an armed anti-poaching team operates within the WMA. Despite this, poaching and retaliatory killings, as well as other acts of resistance against elephant conservation (including property damage), are regularly observed and documented (Nelson, 2007). Compensation payments have been promised for wildlife-related damage and human deaths but remain to be delivered – contributing to distrust and resentment amongst some residents (Homewood, 2017).

d) Baboon management and urban development in the Cape Peninsula, South Africa

The Cape Peninsula is a biodiversity rich headland bordered by the Atlantic Ocean at the south-western tip of Africa. The city of Cape Town is sandwiched between the ocean and the mountain chain that runs the length of the peninsula, forming part of the Table Mountain National Park. A population of Chacma baboons (*Papio ursinus*) inhabit the Peninsula, living in the protected area and surrounding human-modified landscape, feeding on natural vegetation with opportunistic forays into neighbouring urban and agricultural areas (Hoffman and O’Riain, 2011). A combination of non-lethal control, with lethal control for specific individuals, and aversive conditioning-based management has meant that the population has grown from 360 individuals over 10 troops in 1999 (Kansky and Gaynor, 2000) to approximately 550 individuals across 14 troops by 2019. These baboons represent the rich biodiversity and natural heritage of the peninsula, however increasing baboon numbers, lack of ‘baboon-proofing’ on the urban edge, and access to any highly nutritious human-derived food (agriculture, refuse bins, tourists) has increased both the frequency and likelihood of negative interactions with humans (Kaplan et al., 2011). Disagreements on how best to manage the baboon population have been apparent since the trigger – public outrage over the culling of an entire troop of 18 baboons in 1990, which eventually led to the development of a Baboon Management Plan implemented by the multi-stakeholder Baboon Management Team (BMT) in 1998. Since then the conflict has oscillated, with an initial low observed between 1999 and 2001 following the introduction of a non-lethal management strategy (including diurnal monitoring). However, the program failed to keep baboons out of urban areas and conflict intensity repeatedly reached level 4 between 2002 and 2009. In 2010, a new management protocol allowing for euthanasia of problem individuals to be implemented alongside non-lethal methods was published, which, although led to an overall reduction in negative human-baboon interactions, has resulted in a persistent conflict between management and conservation/welfare interests.

e) Conflict in the Macarena Conservation Area, Colombia

The Macarena Conservation Area, located in the northwest corner of the Colombian Amazon basin, exemplifies a conservation conflict resulting from top-down conservation measures. The area comprises three National Parks separated by restricted-use buffer areas. The Macarena was established as a paper Natural Reserve with no defined boundaries in the 1950s. Lack of clear gazetting led to spontaneous human settlement within the reserve. Several attempts in the 1960s and 1970s to legalise these settlements in the Natural Reserve prompted increased human migration to the area in search of land. However, stringent measures introduced in the 1980s to reduce settlements triggered social unrest. Subsequent negotiations resulted in the establishment of legal limits for three National Parks. However, the legal protection of the area did not prevent illegal settlement from occurring throughout the 1990s, at a time of increasing political conflict, military violence, and retaliatory guerrilla warfare. Although hiatuses in conservation conflict intensity were observed during times of improved political context, a latent conflict persisted over disagreements regarding the boundaries, resource uses, and legal status of human settlements within National Parks. In recent years, the conservation conflict was reignited by the implementation of a regional conservation initiative, the Amazon Vision Program, which aimed to increase conservation measures in the Macarena. Despite attempts to reconcile stakeholder interests, settlers in the Conservation Area refused to participate in proposed conservation projects. In 2018, the conflict once again reached level 5, with involvement of the military, access bans and evictions becoming commonplace. Rather than a species-specific case, the Macarena case demonstrates the challenges of legitimacy of National Parks and the interdependence between conservation conflicts and broader socio-economic processes.

f) Vaquita conservation and fishing in the Gulf of California, Mexico

The vaquita (*Phocoena sinus)* is a critically endangered porpoise endemic to the northern portion of the Gulf of California in Mexico (Rojas-Bracho and Taylor, 2017). The vaquita’s charismatic appearance and rapid demise has attracted international attention, funding and collaboration, escalating the issue beyond its small geographic range. The biggest threat to the vaquita’s existence is unintended bycatch during gillnet fishing, often linked to the highly lucrative international trade in the totoaba fish found in the same waters (Jaramillo-Legorreta et al., 2019). These concerns were expressed by the International Whaling Commission’s Scientific Committee soon after its discovery and formal description in 1958. This prompted a series of assessments of the species’ conservation status, including its categorisation as “Vulnerable” by the International Union for the Conservation of Nature in 1978, then “Endangered” in 1990. Since then, the Mexican government has launched several initiatives aimed at conserving the vaquita, including the creation of a biosphere reserve in 1993 with an additional refuge area in 2005 and a fishing gear replacement programme in 2012 (Rojas-Bracho and Reeves, 2013). An emergency ban was placed on gillnet fishing in 2015 (Jaramillo-Legorreta et al., 2019) and an unsuccessful attempt at captive breeding was trialled in 2017, resulting in the mortality of an adult female (Pennisi, 2017). However, illegal fishing has continued in the protected area resulting in 10 vaquitas killed by gillnets between 2016 and 2019 (Jaramillo-Legorreta et al., 2019). Enforcement carried out by the Mexican navy and the NGO Sea Shepherd has reportedly resulted in physical clashes where fishers have been shot (Kessler, 2015; Stevenson, 2019), resulting in a conflict intensity curve that has repeatedly reached level 5.

g) Goose conservation and farming on Islay

The island of Islay lies off the west coast of the Scottish mainland. A large population of barnacle geese (*Branta leucopsis*) overwinter on the island, grazing on agricultural grassland intended to feed cattle and sheep (Mackenzie 2014). A combination of legal protection from hunting and an increasingly benign climate has meant that the overall wintering goose population has grown from fewer than 20,000 individuals in 1970, to around 50,000 by 2015 (MacKenzie & Shaw 2017). On an island where agriculture represents an important source of income and identity, damage to grazing lands by increasing goose numbers threatens local livelihoods and cultural heritage. Disagreements on how best to manage the goose population have been apparent since the trigger – the Wildlife and Countryside Act introduced in 1981 that affords the species legal protection in the United Kingdom. The subsequent conflict was characterised by the implementation of multiple management policies, which in 1988 (compensation to farmers within designated Special Protection Areas for geese), 1992 (whole island compensation scheme) and 2000 (policy involving compensation payments and culling) led to prolonged periods of level 2 conflict. Following successive reviews of the compensation scheme and the strengthening of goose culling, the conflict has oscillated between level 2 and 3 in recent years. Overall, the intensity trend reflects a conflict characterised by strong discourse and occasional actions, but also successful collaborative actions (MacKenzie & Shaw 2017).

**References**

Moreno‐Zarate, L., Estrada, A., Peach, W., & Arroyo, B. (2020). Spatial heterogeneity in population change of the globally threatened European turtle dove in Spain: The role of environmental favourability and land use. *Diversity and Distributions*. <https://doi.org/10.1111/ddi.13067>

Lormée, H., Barbraud, C., Peach, W., Carboneras, C., Lebreton, J. D., Moreno-Zarate, L. A. R. A., ... & Eraud, C. (2019). Assessing the sustainability of harvest of the European Turtle-dove along the European western flyway. *Bird Conservation International*, 1-16.  <https://doi.org/10.1017/S0959270919000479>

Aiyadurai, A. (2016). ‘Tigers are Our Brothers’ Understanding Human-Nature Relations in the Mishmi Hills, Northeast India. *Conservation and Society*, *14*(4), 305-316.

Nijhawan, S. (2018). *Human-animal relations and the role of cultural norms in tiger conservation in the Idu Mishmi of Arunachal Pradesh, India*. Doctoral dissertation, University College London.

Homewood, K. M. (2017). “They Call It Shangri-La”: Sustainable Conservation, or African Enclosures?. In *The Anthropology of Sustainability* (pp. 91-109). Palgrave Macmillan, New York.

Benjaminsen, T. A., Goldman, M. J., Minwary, M. Y., & Maganga, F. P. (2013). Wildlife management in Tanzania: state control, rent seeking and community resistance. *Development and Change*, *44*(5), 1087-1109. <https://doi.org/10.1111/dech.12055>

Evans, L. A., & Adams, W. M. (2016). Fencing elephants: The hidden politics of wildlife fencing in Laikipia, Kenya. *Land Use Policy*, *51*, 215-228. <https://doi.org/10.1016/j.landusepol.2015.11.008>

Nelson, F. (2007). *Emerging or illusory? Community wildlife management in Tanzania*. IIED.

Nijhawan, S. and Mihu, A. (2020). Relations of Blood: Hunting Taboos and Wildlife Conservation in the Idu Mishmi of Northeast India. *Journal of Ethnobiology*, *40*(2), 149-166.

Hoffman, T. S., & O’Riain, M. J. (2011). The spatial ecology of chacma baboons (Papio ursinus) in a human-modified environment. *International Journal of Primatology*, *32*(2), 308-328. <https://doi.org/10.1007/s10764-010-9467-6>

Kansky, R., & Gaynor, D. (2000). Baboon management strategy for the Cape Peninsula. *Final report, Table Mountain fund project number ZA*, *568*, 149.

Kaplan, B. S., O’Riain, M. J., van Eeden, R., & King, A. J. (2011). A low-cost manipulation of food resources reduces spatial overlap between baboons (Papio ursinus) and humans in conflict. *International Journal of Primatology*, *32*(6), 1397-1412. <https://doi.org/10.1007/s10764-011-9541-8>

Rojas-Bracho, L., & Taylor, B. L. (2017). *Phocoena sinus*. The IUCN Red List of Threatened Species 2017.

Jaramillo-Legorreta, A. M., Cardenas-Hinojosa, G., Nieto-Garcia, E., Rojas-Bracho, L., Thomas, L., Ver Hoef, J. M., ... & Tregenza, N. (2019). Decline towards extinction of Mexico's vaquita porpoise (*Phocoena sinus*). *Royal Society Open Science*, *6*(7), 190598. <https://doi.org/10.1098/rsos.190598>

Rojas-Bracho, L., & Reeves, R. (2013). Vaquitas and gillnets: Mexico’s ultimate cetacean conservation challenge. *Endangered Species Research,* *21*, 77–87. <https://doi.org/10.3354/esr00501>

Pennisi, E. (2017). After failed rescue effort, rare porpoise in extreme peril. *Science,* *358*, 851. 10.1126/science.358.6365.851

Kessler, R. (2015). Vaquita porpoises down to ‘way less than 100,’ Mexican agents shoot fisherman while enforcing new protected area. *Mongabay*. Available from <https://news.mongabay.com/2015/05/vaquita-porpoises-down-to-way-less-than-100-mexican-agents-shoot-fisherman-while-enforcing-new-protected-area/>

Stevenson, M. (2019). Mexican Navy: 1 injured in clash near vaquita reserve. *AP News*. Available from <https://www.apnews.com/64f38c8f796c4071b640e872c49a517f>

McKenzie, R., & Shaw, J. M. (2017). Reconciling competing values placed upon goose populations: The evolution of and experiences from the Islay Sustainable Goose Management Strategy. *Ambio*, *46*(2), 198-209. <https://doi.org/10.1007/s13280-016-0880-8>

McKenzie, R. (2014). Islay Sustainable Goose Management Strategy. Scottish Natural Heritage.
