# Supplementary material for: Measuring the intensity of conflicts in conservation
Source: Conserv Lett. 2021 Jan 11;14(3):e12783. doi: 10.1111/conl.12783 (PMC8365684; doi:10.1111/conl.12783)
Supplement: Supplementary file 2 — TABLE S2 Evidence for conflict levels for each of the case studies [file CONL-14-e12783-s004.docx]

Supporting Information S2. Evidence for conflict levels for each of the case studies.

Table S2.1 – Evidence for conflict levels for each of the case studies. Scientific papers and official decrees cited as evidence can be found in the reference list provided at the end of the document. Links to articles are provided in the table for media evidence. Time steps for which there is no evidence are omitted. Shaded rows indicate the start of a case study.

| **Case study** | **Time step** | **Documented event** | **Stakeholder groups involved** | **Intensity level** | **Collaboration** | **Evidence** |
| --- | --- | --- | --- | --- | --- | --- |
| European turtle dove conservation and hunting management in Spain | 2007 | The European Commission Turtle Dove Management Plan lists the species on Annex 2 of the Birds Directive, which permits hunting while recognising the detrimental effect of hunting on the population. It calls for more evidence of the effects of hunting. | European Union | 3 | 0 | European Commission, 2007 |
|  | 2009 | NGO Ecologistas en Accion call for Regional Governments to ban hunting. | Conservation group | 2 | 0 | <https://www.ecologistasenaccion.org/15259/criticamos-a-medio-ambiente-por-permitir-la-caza-de-la-tortola-europea/> |
|  | 2011 | Regional court prohibits hunting season in Castilla-La Mancha because the species’ population status is poor. | Regional government | 3 | 0 | <https://www.seo.org/2017/08/10/comienza-la-caza-en-media-veda-en-espana-a-pesar-del-declive-de-las-especies-y-de-las-condiciones-ambientales-adversas/> |
|  |  | Local environmental group calls for ban on early hunting season in Madrid region. | Conservation group |  | 0 | <https://entornoescorial.blogspot.com/2011/09/los-problemas-de-la-caza-estival-en.html> |
|  | 2012 | SEO/Birdlife warn the species is in danger of extirpation from Spain. | Conservation group | 2 | 0 | <https://www.republica.com/2012/08/13/la-tortola-europea-y-la-codorniz-en-peligro-de-extincion/> |
|  | 2013 | Hunting group asks for volunteer nest monitors, to provide evidence to allow hunting to be maintained. | - Hunting groups - Scientists | 4 | 1 | <http://www.fecaza.com/sala-de-prensa-hemeroteca/hemeroteca/29-mas-noticias/el-rincon-del-federado/2409-proyecto-tortola-seguimiento-de-nidos> |
|  |  | Environmental groups propose a 5-year hunting ban for the species in the Canary Islands. Hunting groups take a protest against the proposal to the local government, amidst fears that the ban would be extended to the rest of the country. | - Conservation groups - Hunting groups |  | 0 | <https://www.fecaza.com/sala-de-prensa-hemeroteca/hemeroteca/130-noticias-generales/2408-la-federacion-canaria-y-la-rfec-defienden-la-caza-de-la-codorniz-y-la-tortola-en-canarias> |
|  |  | Nest and hunting bag monitoring scheme launched. | Hunting groups |  | 1 | <https://www.fecaza.com/sala-de-prensa-hemeroteca/hemeroteca/130-noticias-generales/2462-cazador-colabora-con-el-proyecto-tortola> |
|  |  | Canary Island Regional Government institutes hunting moratorium. | Regional government |  | 0 | <https://www.seo.org/2013/07/26/valiente-paso-de-canarias-al-no-permitir-este-ano-cazar-tortolas-y-codornices/> |
|  |  | SEO/Birdlife lobbies Canary Island Regional Government for permanent hunting ban. | Conservation groups |  | 0 | <https://www.seo.org/2013/07/26/valiente-paso-de-canarias-al-no-permitir-este-ano-cazar-tortolas-y-codornices/> |
|  | 2014 | Canary Island Regional Government extends hunting ban. | Regional government | 3 | 0 | <https://www.seo.org/2014/07/09/el-gobierno-canario-prohibe-la-caza-de-la-tortola-europea-y-la-codorniz/> |
|  |  | SEO/Birdlife calls for nationwide moratorium on hunting. | Conservation group |  | 0 | <https://www.servimedia.es/noticias/408067>  <https://www.efeverde.com/noticias/seobirdlife-alerta-sobre-el-declive-de-tortola-europea-y-codorniz/> |
|  | 2015 | SEO/Birdlife lobbies Cantabrian Regional Government for hunting moratorium for the following year, to which the government agrees. | - Conservation groups - Regional government | 4 | 1 | <https://www.ecoticias.com/naturaleza/100480/prohibicion-caza-tortola-europea-codorniz>  <https://www.europapress.es/cantabria/noticia-caza-tortola-europea-estara-prohibida-cantabria-no-asi-codorniz-20150218204041.html> |
|  |  | Species listed as “Vulnerable” on the IUCN Red List. | IUCN |  | 0 | <https://www.iucnredlist.org/species/22690419/60008772#assessment-information> |
|  |  | Letter coordinated by SEO/Birdlife and signed by 43 ornithologists calling for a hunting ban. | Conservation groups |  | 0 | <https://www.seo.org/wp-content/uploads/2015/05/20150429-Informe-cient%C3%ADficos-Tórtola1.pdf> |
|  |  | Hunting group complains about changes to the open season. | Hunting groups |  | 0 | <https://www.fecaza.com/sala-de-prensa-hemeroteca/hemeroteca/35-mas-noticias/noticias-valencia/3269-2015-04-15-12-26-21> |
|  |  | European turtle dove chosen as “Bird of the Year” by SEO/Birdlife to highlight plight of the species. | Conservation groups |  | 0 | <https://www.seo.org/2015/05/04/notaprensatortola2015/> |
|  | 2016 | Hunting interest websites warn hunters that SEO/Birdlife is only interested in banning hunting, even though evidence suggests habitat change is the greatest threat to the species. | Hunting groups | 4 | 0 | <https://www.cazavision.com/noticia/caza-menor/seo-birdlife-empenada-prohibir-caza-tortola-151229>  <https://revistajaraysedal.es/seo-birdlife-vuelve-a-proponer-la-prohibicion-de-la-caza-de-la-tortola-europea/> |
|  |  | Hunting federation refuses to contribute to the SEO/Birdlife species action plan because they see it as a means to ban hunting. Also claim that their suggested plan has been ignored by SEO/Birdlife. | Hunting groups |  | 0 | <https://cinegeticat.cat/unac-no-colaborara-con-seobirdlife/?lang=es>  <http://acec-canarias.blogspot.com/2016/03/unac-no-colaborara-con-seobirdlife.html> |
|  |  | Listed as "Vulnerable" in the Spanish Catalog of Endangered Species. | Conservation groups |  | 0 | <https://www.miteco.gob.es/es/biodiversidad/temas/conservacion-de-especies/dictamen-streptophelia-turtur_tcm30-378930.pdf> |
|  |  | Species action plan meeting hosted by SEO/Birdlife with other stakeholders, including hunting groups. | - Conservation groups - Hunting groups |  | 1 | <http://oficinanacionaldecaza.org/2017/01/04/jornadas-de-seo-sobre-la-tortola-comun/> |
|  |  | Ecologistas en Accion publish report on the impacts of hunting in Spain, includes summary of evidence regarding turtle dove declines and highlights general impacts of hunting on wildlife in Spain - estimated 25 million animals killed each year. | Conservation groups |  | 0 | <https://spip.ecologistasenaccion.org/IMG/pdf/informe-impacto-caza.pdf> |
|  | 2017 | Hunting federation complains that SEO/Birdlife-organised action plan meeting was not collaborative, that their proposals for improved habitat management were ignored and hunting ban forced through. | Hunting groups | 2 | 0 | <http://oficinanacionaldecaza.org/2017/01/04/jornadas-de-seo-sobre-la-tortola-comun/> |
|  |  | SEO/Birdlife highlights that hunting bags have nearly doubled between 2006 - 2014 while the population has declined by nearly 30% over the same time period. Accuse the Environment Ministry and Regional Governments of ignoring scientific evidence and caving to vested interests. SEO/Birdlife insists that hunting should be prohibited. | Conservation groups |  | 0 | <https://www.seo.org/2017/08/10/comienza-la-caza-en-media-veda-en-espana-a-pesar-del-declive-de-las-especies-y-de-las-condiciones-ambientales-adversas/> |
|  |  | Hunting federation acknowledge the species decline but claim habitat changes are the result and insist a hunting ban will not improve the species status. | Hunting groups |  | 0 | <https://todomonteria.com/la-prohibicion-la-caza-tortola-no-frenara-declive/> |
|  | 2018 | EC Turtle Dove Management Plan - Hunting moratorium proposed in the plan is rejected by Spain, other EU countries, and Federations of Associations for Hunting and Conservation. | - European Union - Conservation groups - Hunting groups | 4 | 0 | <https://ec.europa.eu/environment/nature/conservation/wildbirds/hunting/docs/20181002%20Final_draft_European%20Turtle-Dove.pdf> |
|  |  | Species action plan designed by SEO/Birdlife and the Spanish government. Plan approved by the EC. Includes a hunting moratorium to allow population levels to recover. | - Conservation groups - Spanish government |  | 1 | <https://www.seo.org/2018/05/24/la-tortola-europea-ya-tiene-plan-de-accion/> |
|  |  | Hunting groups complain that hunting has minimal effect on the species, so that the moratorium will be ineffective, and upset rural communities. They argue efforts should be aimed at improving habitats instead. | Hunting groups |  | 0 | <http://www.fecaza.com/sala-de-prensa-hemeroteca/hemeroteca/130-noticias-generales/4134-la-rfec-alerta-de-la-intencion-de-europa-de-apoyar-una-moratoria-de-la-caza-de-la-tortola-que-sera-ineficaz-para-la-recuperacion-de-la-especie>  <http://www.fecaza.com/sala-de-prensa-hemeroteca/hemeroteca/130-noticias-generales/4144-la-prohibicion-de-la-tortola-el-negocio-de-seo-birdlife-y-sus-socios-en-europa> |
|  |  | Complaint that multiple changes to the hunting regulations have had no effect on the numbers of turtle doves being shot. | Scientists |  | 0 | Moreno-Zarate et al (2018) conference poster <https://digital.csic.es/bitstream/10261/175499/1/limiteuropturtle.pdf> |
|  | 2019 | SEO/Birdlife complains to the government about the lack of progress for the species and pressures for an outright ban on hunting. | Conservation groups | 4 | 0 | <https://www.europapress.es/sociedad/medio-ambiente-00647/noticia-seo-birdlife-pide-prohibir-caza-tortola-marco-plan-gestion-sostenible-especie-largo-plazo-20190213164348.html> |
|  |  | European Commission sends formal letters to Spain for breaching Articles 3, 4 and 7 of the Birds Directive, threatening the government with the European Court of Justice, unless Spain takes measures to improve species protection. | European Union |  | 0 | <https://www.euroweeklynews.com/2019/07/30/115664/#.Xd1HTS-cYWo>  <https://www.theguardian.com/environment/2019/jul/25/eu-acts-to-protect-future-of-bird-facing-extinction-in-uk> |
|  |  | 13 Regional governments defy EU and allow hunting. | Regional governments |  | 0 | <https://www.euroweeklynews.com/2019/08/22/spain-has-become-a-deathtrap-for-the-endangered-turtle-dove/#.Xd1F8S-cYWo> |
|  |  | Valencia government (one of the 13 regions that had defied the EU) imposes hunting moratorium for the season, in response to declining hunting bags. | Regional government |  | 0 | <https://www.levante-emv.com/comunitat-valenciana/2019/11/13/fiscalia-insta-prohibir-caza-tortola/1944157.html> |
|  |  | Hunting groups propose a species recovery plan focused on habitat restoration, which will still allow some hunting to take place. | Hunting groups |  | 0 | <http://www.fecaza.com/sala-de-prensa-hemeroteca/hemeroteca/130-noticias-generales/4598-la-rfec-junto-a-otras-entidades-y-cientificos-suscribe-un-manifiesto-a-favor-de-la-conservacion-de-la-tortola-y-apuesta-por-una-gestion-cinegetica-sostenible> |
|  |  | Environment Unit of the State Attorney General's office writes a letter to the 12 regional governments that still allow hunting. | Spanish Government |  | 0 | <https://www.levante-emv.com/comunitat-valenciana/2019/11/13/fiscalia-insta-prohibir-caza-tortola/1944157.html>  <https://elpais.com/sociedad/2019/11/11/actualidad/1573488689_614120.html> |
|  |  | Hunting federations complain that the central government is siding with conservationists, and rejecting hunting groups' species recovery plan. | Hunting groups |  | 0 | <http://www.fecaza.com/sala-de-prensa-hemeroteca/hemeroteca/130-noticias-generales/4640-la-rfec-denuncia-la-amenaza-de-la-fiscalia-a-las-ccaa-sobre-la-tortola-y-reclama-al-miteco-medidas-urgentes-de-gestion-para-la-especie> |
| Tiger conservation, dam development and local livelihoods in Dibang Valley, India | 1998 | Dibang Wildlife Sanctuary (DWLS), covering nearly half of Dibang Valley's area, is established without any public consultation. | - Indian government - Government of the state of Arunachal Pradesh | 4 | 0 | <http://arunachalforests.gov.in/dibang_wildlife_sanctuary.html>  Aiyadurai, 2016 |
|  | 1999-2011 | Little to no enforcement on the ground, but local people uneasy about the existence of the DWLS and its future implications | Local communities | 1 | 0 | Aiyadurai, 2016 |
|  | 2012 | Two orphaned tiger cubs are rescued by conservation NGOs and Indian Government's Forest and Wildlife Department (FD) from a dry well in a village outside DWLS, the tigress died from a snare placed for crop-raiding wild pigs. Dibang begins to draw attention from wildlife researchers, conservation groups and the forest and wildlife department. | - Conservation groups - Forest and Wildlife Department | 3 | 0 | Aiyadurai, 2016  <https://indianexpress.com/article/opinion/editorials/the-besieged-sanctuary-arunachal-tiger-cubs-5501244/>  <http://archive.indianexpress.com/news/two-tigers-in-a-boat-to-say-nothing-of-the-900-km/1181860/> |
|  |  | Local people start to feel abandoned in favour of wildlife. | Local communities |  | 0 |  |
|  | 2013 | Plans to reintroduce tiger cubs to Dibang Valley | Forest and Wildlife Department | 4 | 0 | Aiyadurai, 2016  <https://indianexpress.com/article/opinion/editorials/the-besieged-sanctuary-arunachal-tiger-cubs-5501244/> |
|  |  | Notice issued to village councils to create eco-sensitive zones around DWLS, which would further restrict local land use around the reserve. No consultations are held with local people who are concerned that they will lose more land to DWLS. | Forest and Wildlife Department |  | 0 |  |
|  |  | Opposition to tiger re-introduction | Local communities |  | 0 |  |
|  |  | Local people file a Public Interest Litigation (PIL) in local courts to get more information about the notification of eco-sensitive zones. | Local communities |  | 0 | Aiyadurai, 2016 |
|  | 2014 | Local people do not allow a survey team from an Indian government research institute to enter DWLS to place camera traps for tigers. | Local communities | 3 | 0 |  |
|  | 2015 | Indian government-affiliated research institute begins field research on tigers in DWLS | - Indian government - Affiliated researchers | 3 | 0 |  |
|  |  | Local people express concern at tiger research | Local communities |  | 0 |  |
|  | 2017 | Indian Government's Forest advisory committee does not grant clearance to a large dam in Dibang Valley based on contradictory evidence provided by dam proponents and independent researchers on presence of tigers and other wildlife in community-owned forests. | - Indian government - Independent researchers | 4 | 0 | Nijhawan, 2018 |
|  |  | Some local people file a court appeal questioning the basis for the declaration of DWLS and demanding its area be reduced. The Appeal is supported by dam developers who would like to expand their operations. | - Local communities - Dam developers |  | 0 |  |
|  |  | Government representatives visit Dibang Valley. Local people meet the representatives and express opposition against any proposals for a tiger reserve. | - Forest and Wildlife Department - Local communities |  | 1 |  |
|  |  | Local people concerned about plans to convert DWLS into an exclusive tiger reserve with much stricter access regulations. | Local communities |  | 0 |  |
|  | 2018 | Government-affiliated researchers publish their findings on tigers and promote the conversion of DWLS into a tiger reserve. The study contradicts earlier research by independent researchers that found tigers widespread in Dibang Valley outside of DWLS. | - Government-affiliated researchers - Indian government | 3 | 0 | Adhikarimayum and Gopi, 2018  <https://www.hindustantimes.com/india-news/arunachal-tribe-against-tiger-reserve/story-40UTJv4W8Y3cu1713faA3L.html> |
|  |  | Local communities express opposition to the tiger reserve. | Local communities |  | 0 | <https://www.hindustantimes.com/india-news/arunachal-tribe-against-tiger-reserve/story-40UTJv4W8Y3cu1713faA3L.html> |
|  | 2019 | Community representatives write a letter to the Government of India contesting the conclusions of the research institute's publication, arguing that since their culture has protected tigers, they must be consulted before any future conservation plans are implemented | Local communities | 4 | 0 | Nijhawan, S. (pers. com.)  Aiyadurai. 2020 |
|  |  | Local people hold a meeting to discuss the creation of a tiger reserve. Powerful pro-dam local leaders see tiger presence as a hindrance, blame independent researchers for blocking development in Dibang. | Local communities |  | 0 |  |
|  |  | Decision made to ban all outsiders from conducting wildlife research in Dibang Valley until the boundaries of DWLS are re-demarcated. | Local communities |  | 0 |  |
|  |  | Independent researchers organise a seminar in an attempt to mend relations between researchers and local people. Pro-dam local people voice concerns over the researchers’ portrayal of Dibang Valley as a haven for biodiversity, blame them for blocking developmental activities. | - Local communities - Independent and government-affiliated researchers |  | 1 |  |
| Wildlife area management and local livelihoods in Enduimet, Tanzania | 1997 | Proposals to establish Enduimet Wildlife Management Area - WMAs intended to decentralise wildlife management, proposed as community-run conservation areas. | National Parks Authority | 3 | 0 | Benjaminsen and Bryceson, 2012  Benjaminsen et al., 2013 |
|  | 1998 | Concerns expressed at community village meetings | Local communities | 1 | 0 | Benjaminsen and Bryceson, 2012  Benjaminsen et al., 2013 |
|  | 1999 | Concerns expressed at community village meetings | Local communities | 1 | 0 |  |
|  | 2000 | Disagreement between hunting company and wildlife tour operator over access to land in the region. | - Hunting company - Tourism operator | 2 | 0 | Nelson, 2004 |
|  | 2001 | Concerns expressed at community village meetings | Local communities | 1 | 0 |  |
|  | 2002 | Planning for Enduimet WMA begins in earnest. Formation of community-based organisation (CBO). | - Local communities - Local government - National Parks Authority | 4 | 1 | Nelson, 2007 Trench et al., 2009 |
|  |  | Local residents confront hunting operator and threaten tourists, because they were hunting too close to the village. | - Local communities - Tourists - Hunting company |  | 0 | <https://www.theeastafrican.co.ke/news/ea/4552908-240096-futuc7/index.html> |
|  |  | Local residents view wildlife favourably as a source of tourism revenue but disagree about use of these funds. | Local communities |  | 0 | Nelson, 2004 |
|  | 2003 | Baseline study for proposed pilot WMA. Distrust and disagreement expressed between villages and the National Parks authority over proposed WMA. | - Local communities - Local government - National Parks Authority | 2 | 1 | Kulindwa et al., 2003 |
|  |  | Many local people feel disenfranchised regarding the establishment of the WMA, that the decision to join the WMA was taken by local leaders or government officials, without input from other residents. | Local communities |  | 0 | Minwary, 2009 |
|  | 2004 | Contested land-zoning plan, loss of tourism income, and imposition of trophy hunting. | Local communities | 2 | 0 | Nelson ,2007  Honey, 2008  Trench et al., 2009  Wright, 2016 |
|  | 2005 | Continued contest over land-zoning plan; trophy hunting zone around Sinya village | Local communities | 2 | 0 | Wright, 2016 |
|  |  | Disagreement between hunting company and wildlife tour operator over access to land. | Tourist companies |  | 0 | Minwary, 2009 |
|  |  | Erosion of support for WMA among villages - fears of land alienation, loss of traditional rights, lack of transparency regarding community representatives on the CBOs. | Local communities |  | 0 | Nelson, 2007  Benjaminsen et al., 2013 |
|  | 2006 | 300 acres of cultivation in Tinga Tinga village, but all crops destroyed by elephants. Local residents stated that they no longer tried to grow crops because of crop raiding. | Local communities | 3 | 0 | Minwary, 2009 |
|  |  | Defacement of WMA boundary markers. | Local communities |  | 0 | Nelson, 2007 |
|  | 2007 | Human deaths and injuries from interactions with elephants in MWA. | Local communities | 4 | 0 | Minwary, 2009 |
|  |  | Government revises Wildlife Policy - emphasises state management of wildlife resources over local participation. However, does not include a compensation scheme for wildlife impacts. | Tanzanian government |  | 0 | United Republic of Tanzania, 2007  Benjaminsen et al., 2013 |
|  |  | Establishment of nine-village WMA supported by NGO AWF. | - Local communities - Conservation groups - National Parks Authority |  | 1 | Minwary, 2009  Benjaminsen et al., 2013  Wright, 2017 |
|  |  | Sinya village refuse to join WMA because they have the most wildlife and do not want to share their tourism income, or risk imposition of grading controls. Anti-poaching patrol units also established. | Local communities |  | 0 | Nkwame, 2007  Benjaminsen et al., 2013 |
|  |  | Government forces a wildlife tour operator to move its base of operations from Sinya to another village because Sinya resists joining the WMA. But the tour operator still uses Sinya lands. | Tanzanian government |  | 0 | Benjaminsen et al., 2013  Minwary, 2009 |
|  | 2008 | Disruption of tourism activities by one village - cattle grazing too close to tourist camp, locals blocking tourist vehicle. Camp manager complains. | Local communities | 4 | 0 | Benjaminsen et al., 2013 |
|  |  | Change in policy means that villages no longer receive tourism income from safari operators. Instead, the revenue goes directly to the government. | Tanzanian government |  | 0 | Benjaminsen et al., 2013  Minwary, 2009 |
|  |  | Local residents unhappy about lack of compensation for crop and livestock losses. The government has banned killing of wildlife but offers no compensation for impacts. Compensation is explicitly prevented by the Wildlife Conservation Act. | Local communities |  | 0 | Minwary, 2009 |
|  |  | Local residents concerned about losing access to grazing land at the expense of wildlife. | Local communities |  | 0 |  |
|  |  | Local residents complain about the functioning of the WMA, and their lack of involvement in the decision process. However, the CBO leaders blamed other local figures for not organising the meetings where information is disseminated. | Local communities |  | 0 |  |
|  |  | At the same time, few local residents feel that wildlife brings them any benefit. Most of those people who do feel there is some benefit are directly involved with a tourism operator. | Local communities |  | 0 |  |
|  |  | Some local villages do see benefit of the WMA though, with the distribution of tourism revenue to those villages without much wildlife or tourism operators. Local leaders thought that this would help to alleviate some of the human-wildlife impacts related to crop losses. | - Local communities - Conservation organisations |  | 1 |  |
|  | 2009 | Under pressure from the government and the NGO AWF, the remaining village, Sinya re-joins WMA. | - Local communities - Conservation groups - Tanzanian government | 3 | 1 | Benjaminsen et al., 2013 Wright, 2016 |
|  |  | Member villages of the WMA negotiate a new land-zoning plan and successfully removedthe inhibiting grazing restrictions of the previous plan. | - Local communities - National Parks Authority |  | 1 | Wright, 2017 |
|  |  | Elephant killed in retaliation for trampling a cow. | Local communities |  | 0 | Benjamisen and Svarstad, 2010 |
|  | 2010 | New NGO takes over WMA operations | Conservation groups | 3 | 0 | <https://www.honeyguide.org//wp-content/uploads/2017/11/HG_Strategy_2017_Final.pdf> |
|  | 2011 | Local people concerned about animals destroying their crops. | Local communities | 4 | 0 | Sulle et al., 2011 |
|  |  | Illegal killing of wildlife. | Local communities |  | 0 | Wright, 2016  <https://allafrica.com/stories/201107110646.html> |
|  |  | Trophy hunting operator evicted after court battle. | - Local communities - Hunting company |  | 0 |  |
|  | 2012 | Continued operations in WMA including anti-poaching patrols. | - Local communities - Conservation NGOs - National Parks Authority | 2 | 1 | <https://www.honeyguide.org//wp-content/uploads/2017/11/HG_Strategy_2017_Final.pdf> |
|  |  | Complaints about elephant crop-raiding | Local communities |  | 0 | Wright, 2016 |
|  | 2013 | Increased anti poaching efforts - 40 poachers arrested and no elephants poached. | - Local communities - Conservation groups - National Parks Authority | 0 | 1 | <https://allafrica.com/stories/201302060154.html>  Homewood, 2017  <https://www.honeyguide.org/news/enduimet-zero-elephants-poached-in-2013/> |
|  |  | Firearms training for WMA rangers | - Conservation groups - National Park Authority |  | 1 | <https://www.honeyguide.org/news/firearms-training-in-enduimet/> |
|  |  | Anti-poaching training for WMA rangers | - Conservation groups - National Park Authority |  | 1 | <https://www.honeyguide.org/news/us-security-specialist-trains-wma-rangers/> |
|  |  | Chilli bombs distributed to villagers to help deter elephants from crops. | - Local communities - Conservation groups |  | 1 | <https://www.honeyguide.org/news/making-spirits-bright-with-chili-bombs/> |
|  | 2014 | Elephant poachers arrested in WMA | - Conservation groups - National Park Authority | 3 | 1 | <https://www.honeyguide.org/news/poachers-nabbed-in-west-kilimanjaro/> |
|  |  | Drop in poaching rates but human death via elephant and resistance to one tourism operator and eviction notice to another. | Local communities |  | 0 | Homewood, 2017  <https://allafrica.com/stories/201406300336.html> |
|  |  | Rangers and game scouts attend "Elephant March" calling for an end to poaching and trophy hunting. | - Local communities - Conservation NGOs |  | 1 | <https://www.honeyguide.org/news/march-for-elephants-on-nyerere-day-2/> |
|  |  | Elephant poisoned as retaliation for local crop raiding. | Local communities |  | 0 | <https://www.honeyguide.org/news/october-ends-with-flurry-of-poaching-incidents-arrests/> |
|  | 2015 | "Human-Elephant Conflict Toolkit" developed by NGOs, to train rangers and community volunteers on how to safely deter elephants from raiding farms. | - Local communities - Conservation NGOs | 4 | 1 | <https://www.honeyguide.org/news/partnership-promises-to-strengthen-conservation-communities/>  <http://www.honeyguide.org/wp-content/uploads/2017/11/Honeyguide%20Human%20Elephant%20Conflict%20Handbook%20-%20English%20FINAL%20PRINT.pdf> |
|  |  | Court case over tourism operator. | - Local communities - Tourist company |  | 0 | Wright, 2016 |
|  | 2016 | Retaliatory killing of elephants. | Local communities | 3 | 0 | Baynham-Herd, 2020 |
|  |  | Mitigation efforts continue, with 169 elephant crop-raids mitigated | - Conservation groups - Local communities |  | 1 | <https://s3.amazonaws.com/cms-biglife/files/attached_files/73/BigLife_annualreport_2016_final.pdf> |
|  | 2017 | Illegal grazing of livestock in WMA conservation area. | Local communities | 3 | 0 | <https://www.nation.co.ke/counties/taita-taveta/poaching-Kenya-Tanzania-border/1183326-3807510-8uxyle/> |
|  | 2018 | Local people still feel their concerns regarding the dangers of elephants and crop raiding are being ignored or not fully addressed. | Local communities | 2 | 0 | Dekker, 2018  Baynham-Herd, 2020 |
| Baboon management in Cape Peninsular, South Africa | 1990 | Culling of 18 baboons in the Kommetjie area of the Cape Peninsula raises questions about the management of human-baboon interactions | Local authorities | 3 | 0 | Koutstall, 2013 |
|  | 1991 | Kommetjie Environmental Awareness Group (KEAG) created to promote non-lethal management rather than culling of baboons | - Local residents - Scientists | 3 | 1 | <https://keag1.wordpress.com/about/> |
|  |  | Continued culling of baboons by local authorities in response to resident complaints | Local authorities |  | 0 | Beamish, 2010  Koutstall, 2013  O’Riain 2015 |
|  | 1992 - 1997 | Human-induced mortality of baboons (intentional and accidental) | Local residents | 3 | 0 |  |
|  |  | Continued culling of baboons by local authorities in response to resident complaints | Local authorities |  | 0 |  |
|  | 1998 | Baboon population census finds skewed sex ratio and unsustainable human-induced mortality | Scientists | 3 | 0 |  |
|  |  | Report published advising against baboon culling | Scientists |  | 0 | Kansky and Gaynor, 1998  Koutstall, 2013 |
|  |  | Creation of a Baboon Management Plan (BMP) and Baboon Management Team (BMT) | - Government authorities - Local residents - Conservation organisations - Scientific consultants |  | 1 | Koutstall, 2013  O’Riain, 2015 |
|  | 1999 | Baboon monitors program launched to study behaviour and minimise baboon use of urban areas | - Government authorities - Local residents - Conservation organisations - Scientific consultants | 2 | 1 | Kansky and Gaynor, 2000 |
|  |  | Continued unease of local residents at presence of baboons in urban areas | Local residents |  | 0 | Beamish, 2010  Koutstall, 2013 |
|  | 2000-2001 | Continued unease of local residents at presence of baboons in urban areas | Local residents | 2 | 0 | <https://www.iol.co.za/news/south-africa/baboon-intruders-killed-by-pet-dogs-90274> |
|  |  | Internal polemics and disagreements paralyse the BMT | BMT |  | 0 | Koutstall, 2013  O’Riain, 2015 |
|  | 2002 | Report published to guide local residents and visitors | Scientists | 4 | 0 | Kansky, 2002 |
|  |  | Complaint filed about dysfunctionality of BMT | Scientists |  | 0 | Koutstall, 2013 |
|  |  | Scientists pull out of BMT | Scientists |  | 0 |  |
|  |  | CapeNature takes over management of BMT | Conservation organisation |  | 0 |  |
|  |  | NGO Baboon Matters takes over monitors program | Conservation organisation |  | 0 |  |
|  | 2003 | Baboon tracking via cell phones and satellite collars put into place | BMT | 2 | 1 | <https://www.news.uct.ac.za/article/-2003-11-17-cellphone-technology-comes-to-the-aid-of-peninsulas-baboons> |
|  |  | Continued unease of local residents at presence of baboons in urban areas | Local residents |  | 0 | Beamish, 2010  Koutstall, 2013  O’Riain, 2015 |
|  |  | Internal polemics and disagreements paralyse the BMT | BMT |  | 0 |  |
|  | 2004 | Continued unease of local residents at presence of baboons in urban areas | Local residents | 2 | 0 |  |
|  |  | Internal polemics and disagreements paralyse the BMT | BMT |  | 0 |  |
|  | 2005 | Residents express mixed feelings about baboon management. | Local residents | 3 | 0 | <https://mg.co.za/article/2005-04-01-spoilers-target-baboons> |
|  |  | Local residents injure and kill four baboons. | Local residents |  | 0 | <https://mg.co.za/article/2005-06-20-baboons-the-losers-in-war-with-humans> |
|  |  | Internal polemics and disagreements paralyse the BMT | BMT |  | 0 | Koutstall, 2013 |
|  | 2006 | Creation of the University of Cape Town Baboon Research Unit | - Scientists - BMT | 3 | 1 | O’Riain, 2015 |
|  |  | Concerns after baboon attack on boy and adult man. | Local residents |  | 0 | <https://www.iol.co.za/news/south-africa/brutal-baboon-attacks-raise-concern-266182> |
|  |  | Picnic area fenced off due to baboon presence | Local authorities (Cape Point) |  | 0 | <https://www.capetownmagazine.com/cafes/baboons-on-the-side-cape-points-tasty-lunch-section/93_22_1620> |
|  |  | Capture of baboon raiding in busy neighbourhood | BMT |  | 1 | <https://www.iol.co.za/news/south-africa/wily-baboon-holes-up-in-cape-town-suburb-283099> |
|  |  | Concern over speeding cars hitting and maiming baboons | Conservation organisations |  | 0 | <https://www.iol.co.za/news/south-africa/baboon-baby-lives-after-forest-tragedy-287477> |
|  |  | Concerns voiced about baboon behaviour after British tourist attacked. | Local residents |  | 0 | <https://www.news24.com/SouthAfrica/News/Baboon-attacks-UK-tourist-20061025> |
|  |  | Internal polemics and disagreements paralyse the BMT | BMT |  | 0 |  |
|  | 2007 | Continued concerns about baboon presence following negative encounters | Local residents | 2 | 0 | <https://www.iol.co.za/news/south-africa/baboon-attacks-girl-at-reserve-383252> |
|  |  | Internal polemics and disagreements paralyse the BMT | BMT |  | 0 |  |
|  | 2008 | Baboon caught on a fishing vessel, marked and released. | BMT | 3 | 0 | <https://www.iol.co.za/news/south-africa/fugitive-john-wayne-caught-384496> |
|  |  | Baboon caught and tranquilised after roaming the streets of Cape Town. | BMT |  | 0 | <https://www.independent.co.uk/news/world/africa/law-catches-up-with-john-wayne-cape-towns-celebrity-baboon-770206.html> |
|  |  | Concerns raised over tracking collars making baboons switch behaviour. | Local residents |  | 0 | <https://allafrica.com/stories/200808200052.html> |
|  |  | Internal polemics and disagreements paralyse the BMT | BMT |  | 0 | O’Riain, 2015 |
|  | 2009 | Concerns raised over tourist and baboon behaviour | - Local residents - BMT | 4 | 0 | <https://www.pressreader.com/south-africa/cape-times/20090316/281483567299088> |
|  |  | Concerns over baboons raiding vehicles. | Local residents |  | 0 | <https://www.pressreader.com/south-africa/cape-times/20090330/281479272340874> |
|  |  | Disagreement over best methods to manage baboon problem. | - Local residents - BMT |  | 0 | <https://www.pressreader.com/south-africa/cape-times/20090703/282050503053253> |
|  |  | Walks organized to sensitise people to baboon behaviour. | - Conservation organisations - Local residents |  | 1 | <https://www.jenmansafaris.com/the-cape-town-chacmas-walking-with-baboons/> |
|  |  | Whips introduced as an auditory baboon deterrent, but misuse by residents causes concern for baboon welfare. | - BMT - Local residents - Conservation organisations |  | 0 | <https://www.iol.co.za/news/south-africa/uproar-over-baboon-sjamboks-458301> |
|  |  | Dispute regarding management of tourism, baboon proofing and monitoring amongst stakeholders. | - Local residents - BMT - Tourists |  | 0 | <https://www.pressreader.com/south-africa/sunday-times/20090920/281891589317342> |
|  |  | Baboons cause fear in local residents. | Local residents |  | 0 | <https://www.pressreader.com/south-africa/sunday-times/20090927/281874409453189> |
|  |  | Maps developed to track baboon movement. | Scientists |  | 0 | <https://www.news.uct.ac.za/article/-2009-11-27-no-more-monkey-business-with-baboons> |
|  |  | Internal polemics and disagreements paralyse the BMT | BMT |  | 0 | Koustall, 2013  O’Riain, 2015 |
|  | 2010 | BMT is replaced by two new organisations: the Baboon Technical Team (BTT) and the Baboon Liaison Group (BLG) | - Government authorities - Local residents - Conservation organisations - Scientific consultants | 4 | 1 | Koustall, 2013  O’Riain, 2015 |
|  |  | BTT publishes new “Protocol for reducing the frequency and severity of raiding behaviour by chacma baboons on the Cape Peninsula, South Africa”. The protocol includes non-lethal management such as scaring, but also a controversial clause on euthanasia. | BTT |  | 1 | <https://www.capenature.co.za/wp-content/uploads/2013/11/Protocol-for-raiding-baboons.pdf> |
|  |  | Concerns over baboons affecting wine industry | Wineries |  | 0 | <https://www.telegraph.co.uk/news/worldnews/africaandindianocean/southafrica/7969313/Drunk-baboons-plague-Cape-Towns-exclusive-suburbs.html> |
|  |  | Wine farms plant vines outside their fences as diversionary feeding for baboons | Wineries |  | 0 | <https://www.telegraph.co.uk/news/worldnews/africaandindianocean/southafrica/7517257/South-Africas-baboons-learn-to-enjoy-pinot-noir.html> |
|  |  | Concerns raised over baboon presence and behaviour | Local residents |  | 0 | <https://www.environment.co.za/environmental-issues-news/baboon-qgangsq-run-wild-in-suburban-south-africa.html> |
|  |  | First baboon to be euthanized under new guidelines | BTT |  | 0 | <https://www.telegraph.co.uk/news/worldnews/africaandindianocean/southafrica/7877840/Rogue-baboon-executed-after-terrorising-South-African-residents.html> |
|  |  | Contrasting views regarding baboon control and euthanasia | Local residents |  | 0 | <https://www.pressreader.com/south-africa/weekend-argus-saturday-edition/20100717/281492157592883>  <https://www.pressreader.com/south-africa/cape-argus/20100716/282445640331632> |
|  |  | City of Cape Town increases budget for managing baboons | - Local authorities - BTT |  | 1 | O’Riain, 2015 |
|  | 2011 | Disagreement regarding euthanasia protocol | - BTT - Conservation organisations - Local residents | 2 | 0 | <https://www.pressreader.com/south-africa/weekend-argus-sunday-edition/20110403/281565172295086>  <https://www.bbc.com/news/world-africa-12869928>  <https://www.iol.co.za/news/infamous-baboon-put-down-1047651>  <https://www.pressreader.com/south-africa/weekend-argus-saturday-edition/20110409/282269546938803>  <https://www.iol.co.za/news/south-africa/western-cape/two-more-baboons-put-down-1104580> |
|  | 2012 | Consultant Human Wildlife Solutions (HWS) joins the BTT | - HWS - BTT | 4 | 1 | Koutstall, 2013 |
|  |  | Concerns over disease transmission between baboons and humans. | - Scientists - Local residents |  | 0 | <https://wwwnc.cdc.gov/eid/> |
|  |  | Paintball guns used to deter baboons. | Local residents |  | 0 | <https://issuu.com/thepeoplespost/docs/peoples_post_false_bay_17_april_2012> |
|  |  | Residents fund building of a fence to keep baboons out. | Local residents |  | 0 | <https://www.iol.co.za/news/south-africa/western-cape/baboons-get-shock-measure-1307672> |
|  |  | Concerns of actions of baboon activists | Local residents |  | 0 | <http://www.baboons.org.za/index.php/2015-04-02-12-21-19/news/item/30-activists-and-their-anthropomorphism-remain-greatest-threat-to-baboons> |
|  |  | Continued disagreement over baboon euthanasia | - Local residents - BTT |  | 0 | <https://www.pressreader.com/south-africa/cape-times/20120803/281883000478573>  <https://www.iol.co.za/news/last-alpha-male-not-standing-1389746>  <https://www.iol.co.za/news/two-raiding-baboons-put-down-1401735>  <http://hwsolutions.co.za/reports/>  <http://hwsolutions.co.za/reports/> |
|  |  | Legal action against the protocol for reducing the frequency and severity of raiding behaviour by Chacma baboons of the Cape Peninsula. | Conservation organisation |  | 0 | <https://www.iol.co.za/news/stop-baboon-killings-urge-conservationists-1376275> |
|  |  | Protest by Peninsula residents against the shooting of 24 baboons over a period of 18 months. Protest supported by Baboon Matters; concern that little information is provided to the public. | - Conservation organisations - Local residents |  | 0 | Koutstall, 2013  <https://www.pressreader.com/south-africa/cape-times/20121112/281767036496042> |
|  |  | Meeting held over baboon conflict on the Peninsula. Exposes disagreements over management. | - Local residents - BTT - Conservation organisations |  | 1 | <https://www.iol.co.za/news/south-africa/western-cape/showdown-over-baboons-1421348>  <https://www.iol.co.za/news/south-africa/western-cape/capes-baboon-plan-gets-the-nod-1422295>  <https://www.iol.co.za/news/teeth-bared-in-battle-for-baboon-control-1426170> |
|  | 2013 | Six baboon mortalities as a result of human persecution. | Local residents | 3 | 0 | <http://hwsolutions.co.za/reports/>  <https://www.iol.co.za/news/south-africa/western-cape/probe-into-vicious-attack-on-baboon-1500988> |
|  |  | Management program successful at keeping most baboons out of urban area. | BTT |  | 1 | <https://www.timeslive.co.za/news/south-africa/2013-02-06-most-baboons-kept-out-of-cape-town/> |
|  |  | Continued disagreement over baboon euthanasia | - BTT - Local residents - Conservation organisations |  | 0 | <http://hwsolutions.co.za/reports/>  <https://www.dailymail.co.uk/news/article-2383689/Baboon-burglars-How-gang-kleptomaniac-monkeys-terrorises-estate-South-Africa-residents-scared-leave-house.html>  <https://www.enca.com/south-africa/baboons-run-amok-mother-city> |
|  | 2014 | Human persecution of baboons | Local residents | 3 | 0 | <http://hwsolutions.co.za/reports/> |
|  |  | Continued disagreement regarding baboon euthanasia | - BTT - Local residents |  | 0 |  |
|  | 2015 | Human persecution of baboons | - Local residents | 3 | 0 | <http://hwsolutions.co.za/reports/>  <http://www.turtlesa.com/chacmababoons.html> |
|  |  | Continued disagreement regarding baboon management and euthanasia | - BTT - Local residents |  | 0 |  |
|  | 2016 | Continued human persecution of baboons | Local residents | 3 | 0 | <http://hwsolutions.co.za/reports/>  <https://www.falsebayecho.co.za/news/keep-baboons-wild-5062705>  <https://www.falsebayecho.co.za/news/da-gama-troops-horace-put-down-5431964>  <https://b-guided.co.za/living-side-by-side-with-wild-house-raiding-baboons/> |
|  |  | Continued disagreement about baboon management and euthanasia | - BTT - Local residents |  | 0 | <http://hwsolutions.co.za/reports/>  <https://news.nationalgeographic.com/2016/04/160422-baboons-cape-town-conservation-south-africa/> |
|  |  | Baboon proof bins installed | BTT |  | 1 | <https://www.falsebayecho.co.za/news/baboon-proof-bins-5243625> |
|  |  | Residents encouraged to use lockable bins | Local authorities |  | 0 | <https://www.news24.com/SouthAfrica/News/baboon-proof-your-home-city-of-cape-town-warns-20161223> |
|  | 2017 | Continued human persecution of baboons | Local residents | 3 | 0 | <http://hwsolutions.co.za/reports/> |
|  |  | Welfare and conservation group denounces baboon death following fire and inaction by management authority. | Conservation groups |  | 0 | <https://www.falsebayecho.co.za/news/baboons-death-sparks-row-7510488> |
|  |  | Conservation groups criticise problem animal killing | Conservation groups |  | 0 | <https://www.iol.co.za/news/science/novel-collar-could-bring-peace-to-human-baboon-tussles-8669047> |
|  |  | Disagreement continues over baboon management and euthanasia | - BTT - Conservation groups - Local residents |  | 0 | <https://www.outsideonline.com/2231291/frontlines-south-africas-human-vs-baboon-war>  <http://hwsolutions.co.za/reports/>  <https://www.falsebayecho.co.za/news/surprise-put-to-sleep-for-humane-reasons-11822582> |
|  | 2018 | Continued human persecution of baboons | Local residents | 4 | 0 | <http://hwsolutions.co.za/reports/>  <http://www.baboons.org.za/index.php/2015-04-02-12-21-19/news/item/27-baboon-raider-in-tokai-cape-town-8-january-2018>  <http://www.2oceansvibe.com/2018/07/06/tempers-rage-over-constantia-wine-farms-two-a-day-baboon-hunting-permits/>  <https://www.sentinelnews.co.za/news/permission-to-hunt-baboons-15848867>  <https://www.news24.com/SouthAfrica/News/seven-baboons-killed-after-permits-granted-to-constantia-farms-20180707>  <https://www.thetimes.co.uk/article/call-for-wine-boycott-as-tipsy-baboons-are-shot-dead-klein-constantia-buitenverwachting-nl9bppp5x> |
|  |  | Public appeal following euthanasia of baboon; makes point that removing one problem animal does not solve raiding problem as a whole | Conservation organisations |  | 0 | <https://www.constantiabergbulletin.co.za/news/spotlight-on-management-of-raiding-baboons-12783782> |
|  |  | Baboon hunting permits issued to wine farms | - Local authorities - Wine farms |  | 0 | <https://www.heraldlive.co.za/news/2018-07-09-uproar-over-hunting-permits-for-cape-baboons/>  <https://www.news24.com/SouthAfrica/News/seven-baboons-killed-after-permits-granted-to-constantia-farms-20180707> |
|  |  | Online petition created to halt legal baboon shooting on wine farms | Conservation organisations |  | 0 | <https://www.heraldlive.co.za/news/2018-07-09-uproar-over-hunting-permits-for-cape-baboons/> |
|  |  | Protest against shooting of baboons | Conservation organisations |  | 0 | <https://www.iol.co.za/capeargus/news/protest-against-shooting-of-baboons-on-constantia-wine-farms-15954341>  <https://www.falsebayecho.co.za/news/scant-support-for-hunting-of-baboons-15956152> |
|  |  | Protest asking for withdrawal of hunting permits and for increased transparency from management authorities. | Conservation organisations |  | 0 | <https://www.constantiabergbulletin.co.za/news/baboon-protest-16014934>  <https://www.falsebayecho.co.za/news/public-move-in-defence-of-baboons-16155925>  <https://www.news24.com/SouthAfrica/Local/Peoples-Post/baboon-culling-halted-20180730> |
|  |  | Residents complain about baboon collars | Local residents |  | 0 | <https://www.sentinelnews.co.za/news/collared-baboons-outrage-residents-16821709>  <https://www.falsebayecho.co.za/news/collared-baboons-outrage-residents-16794548> |
| Protected areas, human settlements and illegal crop production in Macarena Conservation Area, Colombia | 1965 | Decree establishing the limits of La Macarena Natural Reserve (MNR) | Colombian government | 3 | 0 | <http://www.suin-juriscol.gov.co/viewDocument.asp?id=1590721>  Puentes Casas, 2013 |
|  | 1966-1967 | Hunting and fishing restrictions imposed within MNR | Colombian government | 4 | 0 | Cubides et al., 1989 |
|  |  | Illegal settlement in MNR | Settlers |  | 0 |  |
|  | 1968-1969 | Settlers urge the government to remove settled areas from MNR boundary | Settlers | 3 | 0 | Gantiva and Iregui, 1998  Del Cairo and Montenegro-Perini, 2015  Quintero and Restrepo, 2009  Cubides et al., 1989  Ceron et al., 2018  Puentes Casas, 2013 |
|  |  | Illegal settlement in MNR | Settlers |  | 0 |  |
|  | 1970-1971 | Buffer zone of the reserve is created by a technical committee and reserve boundaries are modified according to local human settlements | Colombian government | 3 | 0 |  |
|  |  | Government grants rights to settlers of MNR | Colombian government |  | 0 |  |
|  |  | Scientists and conservationists urge government to protect Macarena Conservation Area through a series of articles published in *El Tiempo* | - Scientists - Conservation community |  | 1 |  |
|  | 1976-1977 | Government reneges on rights granted to settlers in 1971 (Sentencia 2396) | Colombian government | 4 | 0 |  |
|  |  | Government institutions and researchers meet to design measures and programs to stop settlement in MNR. | - Colombian government - Researchers |  | 1 |  |
|  |  | Illegal settlement in MNR | Settlers |  | 0 |  |
|  | 1978-1979 | Merging of political and conservation conflicts. Start of military and guerrilla activity within MNR. | - Colombian army - Rebel groups | 4 | 0 |  |
|  |  | Growth of settlement in MNR due to illegal crop production | Settlers |  | 0 |  |
|  | 1980-1981 | Illegal settlement and crop production continue | Settlers | 3 | 0 |  |
|  | 1982-1983 | Illegal settlement and crop production continue | Settlers | 4 | 0 |  |
|  |  | Military campaign against settlement and guerrilla activity in the MNR | Colombian government |  | 0 |  |
|  |  | Settlers mobilize and take control of local municipalities | Settlers |  | 0 |  |
|  | 1984-1985 | Reduction of agricultural loans to settlers occupying MNR | Colombian government | 4 | 0 |  |
|  |  | Settlers mobilize and take control of local municipalities | Settlers |  | 0 |  |
|  |  | Illegal settlement and crop production continue | Settlers |  | 0 |  |
|  | 1986-1987 | Negotiations to redefine limits of MNR (“realinderamiento”) | - Colombian government - Settlers - Public institutions - Conservation NGOs | 3 | 1 |  |
|  |  | Illegal settlement and crop production continue | Settlers |  | 0 |  |
|  | 1988-1989 | MNR is converted into a “Special Management Area” (SMA) consisting of National Parks (NPs) and integrated management areas (DMI) | - Colombian government - National University of Colombia | 4 | 0 | Serna, 2003  [www.suin-juriscol.gov.co/viewDocument.asp?ruta=Decretos/1382075](http://www.suin-juriscol.gov.co/viewDocument.asp?ruta=Decretos/1382075)  Puentes Casas, 2013 |
|  |  | Illegal settlement of NPs and crop production continue | Settlers |  | 0 |  |
|  | 1990-1991 | Meetings and negotiations to create plan to implement the SMA on the ground | - Colombian government - Conservation groups - Settler organisations | 3 | 1 | Puentes Casas 2013 |
|  |  | Illegal settlement of NPs and crop production continue | Settlers |  | 0 |  |
|  | 1992-1993 | Strategy to implement the SMA published | Colombian government | 4 | 0 | Gonzáles-Plazas, 2007 (<https://repository.urosario.edu.co/bitstream/handle/10336/3851/Fasc%c3%adculo3.pdf?sequence=1&isAllowed=y>)  WOLA, 2008 (<http://www.mamacoca.org/docs_de_base/Fumigas/WOLA_Chemical_Reactions_February_2008.pdf>)  Puentes Casas, 2013  Mejía, 2016 |
|  |  | Illegal settlement of NPs and crop production continue | Settlers |  | 0 |  |
|  | 1994-1995 | Resolution 001 allowing for chemical spraying of coca and poppy crops on an experimental basis is approved | Colombian government (National Narcotics Council) | 4 | 0 |  |
|  |  | Illegal settlement and crop production continue | Settlers |  | 0 |  |
|  | 1996-1997 | Use of fumigations to reduce illegal crops in targeted areas | Colombian government | 4 | 0 |  |
|  |  | Protests against use of fumigations, which affect legal crop production and conservation areas | Settlers |  | 0 |  |
|  |  | Program to Eradicate Illicit Crops with Chemicals (PECIG) put forward | Colombian government |  | 0 |  |
|  | 1998-1999 | Peace negotiations initiated between the government and the FARC | - Colombian government - Guerrilla groups | 0 | 1 | Puentes Casas, 2013  Ceron et al., 2018 |
|  |  | First of a series of meetings between body in charge of national park management (UAESPNN) and local settler organisations of Macarena | - Colombian government - Settler organisations |  | 1 |  |
|  |  | Launch of Plan Colombia, which promoted aerial spraying (fumigation) and included the search for alternative livelihoods in an attempt to curb illegal crop production within MNP | - Colombian government - US government |  | 1 |  |
|  |  | Designation of a demilitarized zone within the Macarena SMA, essentially handing over control of the majority of the protected area to the FARC | - Colombian government - Guerrilla groups |  | 1 |  |
|  | 2000-2001 | Environmentalists express concern at the increasing density of roads within the demilitarized zone. They also express concern at the aerial spraying of illegal crops, which they argue harms biodiversity. | Conservation community | 2 | 0 | <https://www.eltiempo.com/archivo/documento/MAM-1212412>  <https://www.eltiempo.com/archivo/documento/MAM-973996>  Puentes Casas, 2013 |
|  | 2002-2003 | End of the demilitarized zone and peace negotiations, increase in military presence within Macarena SMA | Colombian government | 4 | 0 | Puentes Casas, 2013 |
|  |  | Aerial spraying of illegal crops within Macarena SMA and NPs following Resolution 0013 | Colombian government |  | 0 | Puentes Casas, 2013  <https://www.tni.org/es/node/11522#1a>  <https://news.mongabay.com/2005/09/cocaine-destroying-rainforest-parks-in-colombia/> |
|  |  | Court orders suspension of fumigation efforts until effectiveness and risks for the environment are tested | Colombian Civil Society |  | 0 | <http://www.mamacoca.org/FSMT_sept_2003/pdf/AccionPopular-Glifosato.pdf>  <https://www.tni.org/es/node/11522#1a> |
|  | 2004-2005 | Launch of the Plan de Manejo Básico (Basic Management Plan) for Macarena NP | - Colombian government (UAESPNN) - Local settler organisations - Conservation organisations | 3 | 1 | UAESPNN, 2005 |
|  |  | Protests against fumigations and militarization of Macarena SMA | Settlers |  | 0 | <https://www.tni.org/files/download/brief28.pdf>  <https://theecologist.org/2006/mar/01/colombias-killing-fields-first-bio-war-21st-century> |
|  | 2006-2007 | Intensification of fumigation efforts | Colombian government | 5 | 0 | <https://www.unodc.org/pdf/research/icmp/colombia_2006_en_web.pdf>  Gonzáles-Plazas, 2007 (<https://repository.urosario.edu.co/bitstream/handle/10336/3851/Fasc%c3%adculo3.pdf?sequence=1&isAllowed=y>)  <https://www.mindefensa.gov.co/irj/go/km/docs/Mindefensa/noticiasold/Noticias/2006/02/C_Erradicacion_manual_en_La_Macarena_seguira_hasta_el_final.html> |
|  |  | Formal complaint lodged by the National Park authority (UASPNN) against the drug policing authorities for breach of environmental laws | - National Parks Authority - Conservation groups |  | 0 | Quintero and Restrepo, 2009 |
|  |  | Social protests against program to eradicate illegal crops | Local settler organisations |  | 0 | <http://www.mamacoca.org/docs_de_base/Fumigas/Historical_review_of_aerial_sr.html> |
|  | 2008-2009 | Second phase of Plan Colombia | Colombian government | 3 | 0 | Ceron et al., 2018 |
|  | 2010-2011 | Third phase of Plan Colombia | Colombian government | 3 | 0 | Ceron et al., 2018 |
|  | 2012-2013 | Initiation of the peace process between the Colombian government and FARC | - Colombian government - FARC/settlers - International governments | 2 | 1 | Sandoval et al., 2020  <https://www.semana.com/nacion/articulo/el-caguan-tras-17-anos-de-las-negociaciones-con-las-farc/454731-3> |
|  |  | Introduction of alternative production programs (APPs) | - Colombian government - National and International Conservation NGOs - Settler organisations |  | 1 | Sandoval et al., 2020  <https://theredddesk.org/countries/initiatives/early-redd-implementation-colombian-amazon> |
|  |  | APPs met with scepticism by local settlers | Settlers |  | 0 |  |
|  | 2014-2015 | Peace negotiations continue | - Colombian government - FARC/settlers - International governments | 0 | 1 | Sandoval et al., 2020 |
|  | 2016-2017 | End of peace negotiations and signing of a peace agreement. | - Colombian government - FARC/settlers - International governments | 2 | 1 | Sandoval et al., 2020 |
|  |  | The conservation sector expresses concern that the liberation of forest controlled by the FSRC will result in increased deforestation. | Conservation NGOs |  | 0 | Reardon, 2018 |
|  |  | Launch of Amazon Vision Program aimed at achieving zero net deforestation following liberation of large swathes of forest from FARC control. | - Colombian government - Local settler organisation - Conservation NGOs |  | 1 | <https://gggi.org/press-release/amazon-vision-launched-in-colombia/>  <https://www.minambiente.gov.co/images/Atencion_y_particpacion_al_ciudadano/consultas_publicas_2015/viceministerio/Descriptivo-Vision-Amazonia-27Nov2015.pdf> |
|  | 2018-2019 | Refusal of settlers within La Macarena to participate in Amazon Vision Program. | Local settler organisation | 5 | 0 | <https://sostenibilidad.semana.com/impacto/articulo/deforestacion-en-el-caqueta-los-campesinos-tumban-la-selva-para-sobrevivir/39149>  <https://maaproject.org/chiribiquete/>  <https://es.mongabay.com/2019/02/deforestacion-en-colombia-2018-tinigua-macarena-chiribiquete/>  <https://www.elespectador.com/noticias/medio-ambiente/prohibir-las-motosierras-una-solucion-la-deforestacion-articulo-741711>  <https://redd.unfccc.int/files/eicdgb_bosques_territorios_de_vida_web.pdf>  <http://www.meta.gov.co/web/blog/iniciaron-los-operativos-de-la-estrategia-%E2%80%9Cburbuja-ambiental%E2%80%9D-para-contrarrestar-la>. |
|  |  | New spatial evidence shows increasing deforestation within the National Park since 2015. | Scientists |  | 0 |  |
|  |  | Scattered interventions of the government to eradicate crops and evict settlers from the National Parks. | Colombian government |  | 0 |  |
|  |  | National policy to control deforestation area is released. | Colombian government |  | 0 |  |
| Vaquita conservation and fishing in the Gulf of California, Mexico | 1975 | Concerns raised by the International Whaling Commission (IWC)’s Scientific Committee over the impact of incidental mortality of vaquita in totoaba fisheries | IWC | 2 | 0 | Rojas-Bracho et al., 2006  <https://iucn-csg.org/vaquita/> |
|  | 1978 | Researchers include vaquita in list of endangered and rare wildlife species of Mexico | Scientists | 4 | 0 | Rojas-Bracho et al., 2006 |
|  |  | Vaquita classified as Vulnerable by IUCN | International stakeholder (IUCN) |  | 0 | IUCN, 1978 |
|  | 1979 | Proposal for sanctuary to protect the vaquita, amongst other species | Mexican government | 4 | 0 | Rojas-Bracho et al., 2006 |
|  |  | Vaquita listed in Appendix I of CITES | International Stakeholder (CITES) |  | 0 |  |
|  | 1985 | Vaquita listed as Endangered under US Endangered Species Act | US government | 3 | 0 |  |
|  | 1990 | Vaquita listed as Endangered by IUCN | International Stakeholder (IUCN) | 3 | 0 |  |
|  | 1992 | Creation of Technical Committee for the Preservation of Totoaba and Vaquita | Mexican government | 3 | 0 |  |
|  | 1993 | Decree creating Biosphere Reserve of the Upper Gulf of California and Colorado River Delta is published, with the aim of protecting the vaquita | Mexican government | 4 | 0 | Rojas-Bracho et al., 2006  <http://www.unesco.org/mabdb/br/brdir/directory/biores.asp?code=MEX+10&mode=all>  DOF, 1993 |
|  |  | Totoaba gill nets banned | Mexican government |  | 0 | Rojas-Bracho et al., 2006 |
|  |  | Vaquita recovery plan is developed by Mexico with support from Marine Mammal Commission | - Mexican government - International stakeholder (Marine Mammal Commission) |  | 1 | Villa-Ramírez, 1993  Rojas-Bracho et al., 2006 |
|  |  | Mexican standard NOM ‐012‐PESC‐1993 to project vaquita and totoaba in the Gulf of California is published | Mexican government (Ministry of Fisheries) |  | 0 | Rojas-Bracho et al., 2006 |
|  |  | Illegal gillnet fishing leading to vaquita deaths | Fishers |  | 0 | CIRVA, 1997 |
|  | 1994 | Publication of Mexican Standard NOM‐059‐ECOL‐ 1994 – Mexico’s first official list of vulnerable and endangered species. Vaquita is listed as Endangered. | Mexican government (Ministry of Environment) | 4 | 0 | Rojas-Bracho et al., 2006  DOF, 1994 |
|  |  | Illegal gillnet fishing leading to vaquita deaths | Fishers |  | 0 | CIRVA, 1997 |
|  | 1995 | Publication of the Management Plan for the Biosphere Reserve of the Upper Gulf of California and Colorado River Delta | Mexican government | 4 | 0 | Rojas-Bracho et al., 2006 |
|  |  | Illegal gillnet fishing leading to vaquita deaths | Fishers |  | 0 | CIRVA, 1997 |
|  | 1996 | Vaquita listed as Critically Endangered by the IUCN | International stakeholder (IUCN) | 4 | 0 | Rojas-Bracho et al., 2006  <https://www.iucnredlist.org/species/17028/50370296#assessment-information>  <https://iucn-csg.org/vaquita/> |
|  |  | Illegal gillnet fishing leading to vaquita deaths | Fishers |  | 0 | CIRVA, 1997 |
|  | 1997 | Establishment and first meeting of International Committee for the Recovery of the Vaquita (Comité Internacional para la Recuperación de la Vaquita, CIRVA) | - Mexican government - Conservation organisations - International stakeholders (scientists) | 3 | 1 | Rojas-Bracho et al., 2006  Rojas and Reeves, 2013  D’Agrosa et al., 2000  <https://www.mmc.gov/priority-topics/species-of-concern/vaquita/>  <https://iucn-csg.org/vaquita/>  CIRVA, 1997 |
|  |  | Illegal gillnet fishing leading to vaquita deaths | Fishers |  | 0 | CIRVA, 1997 |
|  | 1998 | Vaquita is included in SEMARNAP’s Programme of Prioritized Species | Mexican government | 4 | 0 | Rojas-Bracho et al., 2006 |
|  |  | Illegal gillnet fishing leading to vaquita deaths | Fishers |  | 0 | CIRVA, 1999 |
|  | 1999 | Second meeting of CIRVA | - Mexican government - Conservation organisations - International stakeholders (scientists) | 3 | 1 | CIRVA, 1999 |
|  |  | Illegal gillnet fishing leading to vaquita deaths | Fishers |  | 0 | CIRVA, 1999 |
|  | 2000 | National Fisheries Chart is published and establishes that vaquita by‐catch should be zero | National Fisheries Institute (INAPESCA) | 4 | 0 | Alvarez-Torres et al., 2002 |
|  |  | New legislation in Mexico strengthens conservation efforts. The “Rules of the General Law of Ecological Equilibrium and Environmental Protection in matters of Natural Protected Areas” prohibit fisheries that result in by-catch of vaquita, totoaba, sea turtles or any other species at risk in the reserve. | Mexican government |  | 0 | <https://porpoise.org/about-porpoises/vaquita/> |
|  |  | Illegal gillnet fishing leading to vaquita deaths | Fishers |  | 0 | CIRVA, 2004 |
|  | 2001 | Establishment of the Consultative Technical Subcommittee for the Recovery and Protection of the Vaquita. | - International stakeholders (NGOs) - Mexican government | 3 | 1 | SEMARNAT, 2008 (<https://www.gob.mx/cms/uploads/attachment/file/350199/PACE_Vaquita_en_Ingles.pdf>) |
|  |  | Survey of fisher communities reveal desire to protect the vaquita in the long-term. | - Intercultural Center for the Study of Deserts and Oceans - Fishing sector |  | 1 | North American Conservation Action Plan: Vaquita 2008 (<http://www3.cec.org/islandora/en/item/1136-vaquita-north-american-conservation-action-plan>) |
|  |  | Illegal gillnet fishing leading to vaquita deaths | Fishers |  | 0 | CIRVA, 2004 |
|  | 2002 | WWF‐Gulf of California establishes the Joint Initiative with other non‐governmental organizations and CIRVA to promote implementation of CIRVA’s recommendations and develop an economic and legal framework | - WWF - CIRVA - Mexican government | 4 | 1 | Rojas-Bracho et al., 2006 |
|  |  | SEMARNAT publishes Emergency Mexican Official Standard NOM-EM-139-SEMARNAT-2002 prohibiting the use of trawl nets and of trammel and gillnets with a mesh larger than 6 inches within the buffer zone of the Biosphere Reserve. | Mexican government |  | 0 | Rojas-Bracho et al., 2006 |
|  |  | Opposition to NOM-EM-139-SEMARNAT-2002 by fishing sector results in environmental impact authorization allowing 162 trawlers to operate in the Reserve for 3 months. | - Mexican government - Fishing sector |  | 0 | SEMARNAT, 2008  Cantú-Guzmán et al., 2015 |
|  |  | Illegal gillnet fishing leading to vaquita deaths | Fishers |  | 0 | CIRVA, 2004 |
|  | 2003 | Public forums on updating the Reserve Conservation and Management Program | - Mexican government - Mexican NGOs | 3 | 1 | Rojas-Bracho et al., 2006  SEMARNAT, 2008 |
|  |  | Illegal gillnet fishing leading to vaquita deaths | Fishers |  | 0 | CIRVA, 2004 |
|  | 2004 | International Whaling Commission Scientific Committee (IWC-SC) reiterates and endorses CIRVA’s conclusions, calling upon the Mexican Government to implement conservation recommendations | IWC-SC | 3 | 1 | SEMARNAT, 2008 |
|  |  | Third meeting of CIRVA | - Mexican government - International stakeholders (scientists) |  | 1 | CIRVA, 2004 |
|  |  | Illegal gillnet fishing leading to vaquita deaths | Fishers |  | 0 | CIRVA, 2004 |
|  | 2005 | The Sustainable Upper Gulf (AGS) group is formed by representatives of the inshore and industrial fishing sector, the region’s leading shrimp marketing company, and national and international civic organizations involved in conservation | - Shrimp exporter - Fishing sector - National and International NGOs | 3 | 1 | Rojas-Bracho et al., 2006  Cantú-Guzmán et al., 2015  SEMARNAT, 2008 |
|  |  | Creation of the Refuge Area for the Protection of the Vaquita covering 80% of the area in which sightings have occurred | - Mexican government - AGS - CIRVA |  | 1 | <https://porpoise.org/about-porpoises/vaquita/>  Rojas-Bracho et al., 2006  Cantú-Guzmán et al., 2015  DOF, 2005  Rojas-Bracho & Reeves, 2013 |
|  |  | Illegal gillnet fishing leading to vaquita deaths | Fishers |  | 0 | CIRVA, 2012 |
|  | 2007 | President of Mexico announces the Conservation Program for Endangered Species (Programa de Conservación de Especies en Riesgo − PROCER) to implement Species Conservation Action Pro- grams (Programas de Acción para la Conservación de Especies − PACE) for a list of selected species, including the vaquita among the top 5. | Mexican government | 4 | 0 | Rojas-Bracho & Reeves, 2013  <https://iucn-csg.org/vaquita/>  Avila-Forcada et al., 2020  Avila-Forcada et al., 2012  Rojas-Bracho & Fueyo, 2010  SEMARNAT, 2008 |
|  |  | Letter from IUCN Director-General to President of Mexico requesting that his government take all necessary measures immediately to ensure that the vaquita, a national treasure of Mexico, does not go extinct | International stakeholder (IUCN) |  | 0 | Rojas-Bracho & Reeves, 2013 |
|  |  | North American Conservation Action Plan: Vaquita is launched - a cooperative initiative involving scientists, academics, environmental groups, and officials from Canada, Mexico and the USA | - Mexican government - US government - Canadian government |  | 1 | Rojas-Bracho & Reeves, 2013  North American Conservation Action Plan: Vaquita 2008 (<http://www3.cec.org/islandora/en/item/1136-vaquita-north-american-conservation-action-plan>) |
|  |  | Illegal gillnet fishing leading to vaquita deaths | Fishers |  | 0 | CIRVA, 2012 |
|  | 2008 | PACE-Vaquita is presented to stakeholders | Mexican government | 4 | 1 | Rojas-Bracho & Reeves, 2013  Avila-Forcada et al., 2012 |
|  |  | Vaquita survey reveals a population decline of 7.6% per year between 1997 and 2008 | Scientists |  | 0 | Gerodette et al., 2011  Jaramillo-Legorreta et al., 2017 |
|  |  | Illegal gillnet fishing leading to vaquita deaths | Fishers |  | 0 | CIRVA, 2012 |
|  | 2009 | Local fishers present Environmental Impact Assessment to allow them to fish in the protected area | Fishing sector | 4 | 0 | Rojas-Bracho & Reeves, 2013 |
|  |  | Acoustic monitoring scheme is implemented by the National Institute of Ecology | Research institute |  | 0 | Rojas-Bracho & Reeves, 2013 |
|  |  | Illegal gillnet fishing leading to vaquita deaths | Fishers |  | 0 | CIRVA, 2012 |
|  | 2011 | Ship owners present an EIA for blue and brown shrimp fishing in the buffer zone of the Biosphere Reserve | Fishing sector | 4 | 0 | Rojas-Bracho & Reeves, 2013 |
|  |  | Illegal gillnet fishing leading to vaquita deaths | Fishers |  | 0 | CIRVA, 2012 |
|  | 2012 | Fourth meeting of CIRVA | - Mexican government - International stakeholders (scientists) | 4 | 1 | CIRVA, 2012  <https://iucn-csg.org/update-on-conservation-efforts-for-the-vaquita/> |
|  |  | The National Advisory Committee for Responsible Fisheries adopts a 3-year phase-out to remove gillnets in all of the vaquita’s range. | Mexican government |  | 0 | Rojas-Bracho & Reeves, 2013 |
|  |  | Illegal gillnet fishing leading to vaquita deaths | Fishers |  | 0 | CIRVA, 2012 |
|  | 2013 | First meeting of the Comisión Asesora de la Presidencia de México para la Recuperación de la Vaquita (Advisory Commission of the Presidency of Mexico for the Recovery of the Vaquita) | - Mexican government - Natural resource committees - State representatives - Fisheries unions - Mexican Navy - NGOs - Private foundations | 3 | 1 | <https://iucn-csg.org/new-presidential-commission-to-save-vaquita-takes-first-steps/> |
|  |  | Illegal gillnet fishing leading to vaquita deaths | Fishers |  | 0 | CIRVA, 2014 |
|  | 2014 | Mexican presidential commission meets to discuss recent CIRVA report | - Mexican government - Natural resource committees - State representatives - Fisheries unions - Mexican Navy - NGOs - Private foundations | 3 | 1 | <https://iucn-csg.org/the-vaquita-new-report-from-cirva-released/>  CIRVA, 2014  <https://iucn-csg.org/vaquita-conservation-update/> |
|  |  | Illegal fishing in vaquita refuge | Fishing sector |  | 0 | <https://iucn-csg.org/vaquita-conservation-update/>  <https://iucn-csg.org/new-evidence-that-mexican-authorities-are-not-adequately-enforcing-fishing-regulations-to-protect-vaquitas/> |
|  |  | Fifth meeting of CIRVA | - Mexican government - International stakeholders (scientists) |  | 1 | <https://iucn-csg.org/the-vaquita-new-report-from-cirva-released/>  CIRVA, 2014 |
|  | 2015 | Emergency 2-year ban on gillnet fishing in biosphere reserve | Mexican government | 5 | 0 | <https://news.mongabay.com/2016/07/mexico-bans-gillnets-to-protect-rare-vaquita-porpoise/>  Taylor et al., 2017 |
|  |  | The president of the Society of Marine Mammalogy sends a letter to the Mexican president urging action over illegal fishing in the vaquita refuge | International stakeholder |  | 0 | <https://www.marinemammalscience.org/letters/letter-to-president-nieto-regarding-the-vaquita/presidential-letter-vaquita/> |
|  |  | Fisherman shot by navy and taken to hospital. | Mexican navy |  | 0 | <https://news.mongabay.com/2015/05/vaquita-porpoises-down-to-way-less-than-100-mexican-agents-shoot-fisherman-while-enforcing-new-protected-area/> |
|  |  | Illegal gillnet fishing leading to vaquita deaths | Fishers |  | 0 | CIRVA, 2015 |
|  |  | Sixth meeting of CIRVA | - Mexican government - International stakeholders (scientists) |  | 1 | <https://iucn-csg.org/vaquita-decline-even-faster-than-expected/>  CIRVA, 2015 |
|  |  | Operation Milagro launched by the Sea Shepherd Conservation Society to patrol vaquita habitat | NGO |  | 0 | <https://seashepherd.org/campaigns/milagro/> |
|  | 2016 | Illegal gillnet fishing leading to vaquita deaths | Fishers | 3 | 0 | <https://iucn-csg.org/vaquita-update-three-documented-deaths-in-one-month-not-good/>  <https://iucn-csg.org/update-on-the-vaquita/>  CIRVA, 2016 |
|  |  | Seventh meeting of CIRVA | - Mexican government - International stakeholders (scientists) |  | 1 | <https://iucn-csg.org/extinction-is-imminent-new-report-from-vaquita-recovery-team-cirva-is-released/>  CIRVA, 2016 |
|  | 2017 | Illegal gillnet fishing leading to vaquita deaths | Fishers | 4 | 0 | <https://iucn-csg.org/2361/>  CIRVA, 2017 |
|  |  | Eight meeting of CIRVA | - Mexican government - International stakeholders (scientists) |  | 1 | <https://iucn-csg.org/vaquita-on-the-verge-of-extinction/>  CIRVA, 2017 |
|  |  | Permanent ban on gillnet fishing | Mexican government |  | 0 | <https://news.mongabay.com/2016/07/mexico-bans-gillnets-to-protect-rare-vaquita-porpoise/>  <https://edition.cnn.com/2017/07/02/americas/mexico-bans-gill-nets-vaquita-porpoise/index.html>  Pennisi, 2017 |
|  |  | Start and end of captive breeding program (vaquitaCPR) after death of vaquita during capture | - Mexican government (Ministry of Environment) - International scientist coalition |  | 1 | Pennisi, 2017  <https://swfsc.noaa.gov/textblock.aspx?Divisions=PRD&ParentMenuId=678&id=21640>  Rojas-Bracho et al., 2019 |
|  |  | Ninth meeting of CIRVA | - Mexican government - International stakeholders (scientists) |  | 1 | <https://www.iucn-csg.org/wp-content/uploads/2010/03/CIRVA-9-Final-Report-May-11-2017.pdf>  CIRVA, 2017 |
|  |  | Tenth meeting of CIRVA | - Mexican government - International stakeholders (scientists) |  | 1 | CIRVA, 2018 |
|  | 2018 | Totoaba farming program launched to curb illegal fishing | - Mexican government - Fishers | 4 | 1 | CIRVA, 2018 |
|  |  | Mexican troops and Sea Shepherd Conservation Society partner to protect the vaquita | - Mexican navy - International NGO |  | 0 | <https://phys.org/news/2018-03-mexican-troops-partner-activists-vaquita.html> |
|  |  | Illegal gillnet fishing leading to vaquita deaths | Fishers |  | 0 | Jaramillo-Legorreta et al., 2019  CIRVA, 2018 |
|  | 2019 | Vaquita protection boats attacked by fishermen | Fishers | 5 | 0 | <https://www.theguardian.com/environment/2019/jan/10/mexican-fishermen-attack-sea-shepherd-vaquita-porpoise> |
|  |  | Clashes between fishermen and Mexican navy boats in charge of protecting the vaquita | - Mexican navy - Fishers |  | 0 | <https://maritime-executive.com/article/fisherman-shot-during-vaquita-conservation-enforcement> |
|  |  | Illegal gillnet fishing leading to vaquita deaths | Fishers |  | 0 | Flessa et al., 2019 |
|  |  | Eleventh meeting of CIRVA | - Mexican government - International stakeholders (scientists) |  | 1 | <https://iucn-csg.org/wp-content/uploads/2019/03/CIRVA-11-Final-Report-6-March.pdf>  CIRVA, 2019 |
| Goose conservation and farming on Islay, Scotland | 1981 | Wildlife and Countryside Act grants protection to all wild bird species in the UK and sets strict rules for their management on agricultural land | UK government | 3 | 0 | <https://www.gov.uk/guidance/wild-birds> |
|  | 1982 | Start of whole island counts of geese on Islay | SNH | 3 | 0 | McKenzie and Shaw, 2017 |
|  | 1983 | Renotification of three Sites of Special Scientific Interest (SSSI) to allow the development of a sanctuary management strategy | - SNH - Wildfowl and Wetlands Trust | 4 | 0 | McKenzie and Shaw, 2017  Bignal et al., 1991  Owen, 1977  Owen, 1990 |
|  |  | Department of Agriculture and Fisheries for Scotland (DAFS) threatens to issue licences for goose culling within SSSIs if conflict with farmers is not minimised | DAFS |  | 0 | Bignal et al., 1991 |
|  |  | Management agreements drawn with farmers whereby they receive payments and fertilizer to offset the effect of geese. | - SNH - Farmers |  | 1 | McKenzie and Shaw, 2017  Bignal et al., 1991 |
|  |  | The Royal Society for the Protection of Birds (RSPB) acquires Loch Gruinart | RSPB |  | 0 | Whitehouse, 2009 |
|  |  | Non-systematic scaring of geese by farmers | Farmers |  | 0 | Bignal et al., 1991 |
|  | 1984 | Farmers start to express disagreement at the way Loch Gruinart is managed by the RSPB (like a nature reserve and less like a farm) and ask for licences to shoot geese | Farmers | 2 | 0 | Bignal et al., 1991  Whitehouse, 2009 |
|  | 1985 | Farmers express disagreement at the way Loch Gruinart is managed by the RSPB (like a nature reserve and less like a farm) and ask for licences to shoot geese | Farmers | 3 | 0 | Bignal et al., 1991 |
|  |  | Licensed shooting of geese by farmers | Farmers |  | 0 | Percival et al., 1997 |
|  | 1986 | Farmers express disagreement at the way Loch Gruinart is managed by the RSPB (like a nature reserve and less like a farm) and ask for licences to shoot geese | Farmers | 3 | 0 | Bignal et al., 1991 |
|  |  | Licensed shooting of geese by farmers | Farmers |  | 0 | Percival et al., 1997 |
|  | 1987 | Start of systematic scaring scheme aimed at coordinating scaring of geese into feeding areas | - SNH - DAFS - Farmers | 3 | 1 | Bignal et al., 1991  McKenzie and Shaw, 2017  Percival et al., 1991  Ogilvie, 1992  Percival et al., 1997 |
|  |  | Licensed shooting of geese by farmers | Farmers |  | 0 | Percival et al., 1997 |
|  | 1988 | Designation of five Special Protection Areas (SPAs) for geese on Islay. Farmers within SPAs receive financial compensation in return for the provision of good quality grassland for grazing geese | - NCC - Farmers | 2 | 1 | McKenzie & Shaw 2017  Percival et al., 1997 |
|  |  | Farmers outside SPAs complain that geese affect them but they don’t receive compensation | Farmers |  | 0 |  |
|  | 1989 | Farmers outside SPAs complain that geese affect them but they don’t receive compensation | Farmers | 2 | 0 | Ogilvie, 1992 |
|  | 1990 | Farmers outside SPAs complain that geese affect them but they don’t receive compensation | Farmers | 2 | 0 |  |
|  | 1991 | Farmers outside SPAs complain that geese affect them but they don’t receive compensation | Farmers | 2 | 0 |  |
|  | 1992 | Management plan extended to the whole island – all farmers eligible to receive compensation payments for feeding geese | - SNH - Farmers | 2 | 1 | McKenzie and Shaw, 2017  Mason et al., 2017  Ogilvie, 1992 |
|  |  | On-going debate about whether compensation payments are sufficient to cover the cost of geese | Farmers |  | 0 | Percival et al., 1997  Bainbridge, 2017  Cope et al., 2005  McKenzie, 2014 |
|  | 1993 | On-going discussions regarding the effectiveness of the whole-island management plan. | - Farmers - Conservation organisations - SNH | 2 | 0 |  |
|  | 1994 |  |  |  | 0 |  |
|  | 1995 |  |  |  | 0 |  |
|  | 1996 |  |  |  | 0 |  |
|  | 1997 |  |  |  | 0 |  |
|  | 1998 |  |  |  | 0 |  |
|  | 1999 | Creation of the National Goose Forum (NFG) | - SNH - Farmer associations - Conservation organisations - Hunting associations | 2 | 1 |  |
|  |  | On-going discussions regarding the effectiveness of the whole-island management plan | - Farmers - Conservation organisations - SNH |  | 0 |  |
|  | 2000 | Judicial Review decision against licenses issued to shoot barnacle geese in SPAs sought by RSPB and WWT | Conservation organisations | 3 | 0 | McKenzie, 2014  McKenzie and Shaw, 2017  Bainbridge, 2017 |
|  |  | Formation of the National Goose Management Review Group (NGMRG) at the national level and Islay Goose Management Review Group (ILGMRG) on Islay | - SNH - Farmer associations - Conservation organisations - Hunting associations |  | 1 |  |
|  |  | Launch of new goose management scheme on Islay to deliver national goose policy objectives including feeding geese but protecting crops by non-lethal scaring and licensed shooting to protect the most valuable crops | ILGMRG |  | 1 |  |
|  | 2001 | Concern by farmers that the compensation scheme will not cover their losses due to geese. | Farmers | 2 | 0 | McKenzie and Shaw, 2017  Cusack et al., 2018 |
|  |  | Complaints against goose culling | Conservation organisations |  | 0 |  |
|  | 2002 | Concern by farmers that the compensation scheme will not cover their losses due to geese. | Farmers | 2 | 0 |  |
|  |  | Complaints against goose culling | Conservation organisations |  | 0 |  |
|  | 2003 | Concern by farmers that the compensation scheme will not cover their losses due to geese. | Farmers | 2 | 0 |  |
|  |  | Complaints against goose culling | Conservation organisations |  | 0 |  |
|  | 2004 | Concern by farmers that the compensation scheme will not cover their losses due to geese. | Farmers | 2 | 0 |  |
|  |  | Complaints against goose culling | Conservation organisations |  | 0 |  |
|  | 2005 | Review of compensation payments; payment rates are increased but this does not seem to cover the costs incurred from an increasing goose population | National Goose Management Research Group | 2 | 1 |  |
|  |  | Complaints against goose culling | Conservation organisations |  | 0 |  |
|  |  | Concern by farmers that the compensation scheme will not cover their losses due to geese. | Farmers |  | 0 |  |
|  | 2006 | Concern by farmers that the compensation scheme will not cover their losses due to geese. | Farmers | 2 | 0 |  |
|  |  | Complaints against goose culling | Conservation organisations |  | 0 |  |
|  | 2007 | Concern by farmers that the compensation scheme will not cover their losses due to geese. | Farmers | 2 | 0 |  |
|  |  | Complaints against goose culling | Conservation organisations |  | 0 |  |
|  | 2008 | Review of compensation payments; payment rates are increased but this does not seem to cover the costs incurred from an increasing goose population | National Goose Management Research Group | 2 | 1 |  |
|  |  | Concern by farmers that the compensation scheme will not cover their losses due to geese. | Farmers |  | 0 |  |
|  |  | Complaints against goose culling | Conservation organisations |  | 0 |  |
|  | 2009 | Concern by farmers that the compensation scheme will not cover their losses due to geese. | Farmers | 2 | 0 | <https://www.islay.blog/article.php/goose-management-scheme> |
|  |  | Complaints against goose culling | Conservation organisations |  | 0 |  |
|  | 2010 | Cuts to compensation payments | Scottish government | 3 | 0 | Crabtree et al., 2010  McKenzie and Shaw, 2017  <https://www.rspb.org.uk/Images/goosecomplaint_tcm9-407227.pdf> |
|  |  | Concern by farmers that the compensation scheme will not cover their losses due to geese. | Farmers |  | 0 |  |
|  |  | Complaints against goose culling | Conservation organisations |  | 0 |  |
|  | 2011 | Revision of goose management scheme to include weighted payments | Islay Goose Management Group | 2 | 1 | McKenzie and Shaw. 2017  Cusack et al., 2018 |
|  |  | Complaints against goose culling | Conservation organisations |  | 0 |  |
|  | 2012 | Weighted payments dropped | Islay Goose Management Group | 3 | 0 |  |
|  |  | Review of compensation payments, government cap on funding, population control through shooting strengthened | - Scottish government - Islay Goose Management Group - SNH |  | 1 |  |
|  |  | Complaints against goose culling | Conservation organisations |  | 0 |  |
|  | 2013 | Agreement reached to develop an adaptive management scheme that would include controlled culling of the goose population to minimise costs to farmers | - Farmers - SNH - Scottish government - Islay Goose Management Group | 2 | 1 |  |
|  |  | Complaints against goose culling | Conservation organisations |  | 0 |  |
|  | 2014 | Development of a new Islay Goose Management Scheme | - Farmers - SNH - Scottish government - Conservation groups | 2 | 1 | McKenzie and Shaw, 2017  McKenzie, 2014  [https://www.birdguides.com/news/controversial-islay-goose-management-strategy-announced/#](https://www.birdguides.com/news/controversial-islay-goose-management-strategy-announced/)  <https://www.nfus.org.uk/news/news/way-forward-geese-management-charted-national-conference> |
|  |  | Conservation NGOs express concern at the management plan | Conservation groups |  | 0 | [https://www.birdguides.com/news/controversial-islay-goose-management-strategy-announced/#](https://www.birdguides.com/news/controversial-islay-goose-management-strategy-announced/) |
|  | 2015 | New Goose Management Scheme comes into effect. | - SNH - Islay Goose Management Group | 3 | 1 | McKenzie and Shaw, 2017 |
|  |  | Formal complaint to the EC filed by RSPB and WWT. | Conservation groups |  | 0 | <https://www.rspb.org.uk/Images/goosecomplaint_tcm9-407227.pdf>  <https://community.rspb.org.uk/ourwork/b/scotland-directors-blog/posts/the-islay-goose-strategy-and-why-we-have-complained-to-europe> |
|  |  | RSPB and WWT resign from National Goose Management Review Group | Conservation groups |  | 0 | <https://community.rspb.org.uk/ourwork/b/scotland-directors-blog/posts/the-islay-goose-strategy-and-why-we-have-complained-to-europe> |
|  | 2017 | Interim review of the Islay Goose Management Strategy | - SNH - Farmers - Conservation organisations | 2 | 1 | <https://www.parliament.scot/S5_Environment/General%20Documents/National_Goose_Policy_Framework_Interim_review_2017.pdf> |
|  |  | Conservation organisations continue to express concern at goose cull | Conservation organisations |  | 0 |  |
|  | 2018 | Welfare groups call for the “slaughter” of geese on Islay to be stopped. | Animal welfare groups | 2 | 0 | <https://theferret.scot/geese-shooting-islay-scientists/> |
|  | 2019 | Shared videos of geese being shot prompt renewed allegations of animal cruelty. | Animal welfare groups | 3 | 0 | <https://theferret.scot/film-geese-shot-injured-islay/> |

**References**

Adhikarimayum, A. S., & Gopi, G. V. (2018). First photographic record of tiger presence at higher elevations of the Mishmi Hills in the Eastern Himalayan Biodiversity Hotspot, Arunachal Pradesh, India. *Journal of Threatened Taxa*, *10*(13), 12833-12836.

Aiyadurai, A. (2016). ‘Tigers are Our Brothers’ Understanding Human-Nature Relations in the Mishmi Hills, Northeast India. *Conservation and Society*, *14*(4), 305-316.

Aiyadurai. A. (2020). Seminar Report: Initiating dialogue between Idu Mishmi and research scholars. December 10-11, 2019.

Alvarez-Torres, P., Díaz-de-León-Corral, A., Ramírez-Flores, O., & Bermúdez-Rodríguez, E. (2002). National fisheries chart 2000: a new instrument for fisheries management in inland waters. *Reviews in Fish Biology and Fisheries*, *12*(2-3), 317-326.

Avila-Forcada, S., Martínez-Cruz, A. L., & Munoz-Pina, C. (2012). Conservation of vaquita marina in the Northern Gulf of California. *Marine Policy*, *36*(3), 613-622.

Avila-Forcada, S., Martinez-Cruz, A. L., Rodriguez-Ramirez, R., & Sanjurjo-Rivera, E. (2020). Transitioning to alternative livelihoods: The case of PACE-Vaquita. *Ocean & Coastal Management*, *183*, 104984.

Bainbridge, I. (2017). Goose management in Scotland: An overview. *Ambio*, *46*(2), 224-230.

Baynham-Herd, Z. (2020). Behavioural interventions in conservation conflicts. Doctoral thesis, University of Edinburgh. Available at

Benjaminsen, T. A., & Bryceson, I. (2012). Conservation, green/blue grabbing and accumulation by dispossession in Tanzania. *Journal of Peasant Studies*, *39*(2), 335-355.

Benjaminsen, T. A., & Svarstad, H. (2010). The death of an elephant: Conservation discourses versus practices in Africa. *Forum for Development Studies,* *37*(3), 385-408.

Benjaminsen, T. A., Goldman, M. J., Minwary, M. Y., & Maganga, F. P. (2013). Wildlife management in Tanzania: state control, rent seeking and community resistance. *Development and change*, *44*(5), 1087-1109.

Bignal, E. M., Stroud, D. A., & Easterbee, N. (1991). Goose damage and management workshop. Proceedings of a meeting organised by the Wildfowl and Wetlands Trust at Martin Mere, Lancashire, on 27 April 1990. In: Owen, M. & Pienkowski, M.W. (Eds.) (1991). Research & survey in nature conservation No. 33: 22 31. JNCC, Peterborough.

Cantú-Guzmán, J. C., Oliviera-Bonavilla, A., & Sánchez-Saldaña, M. E. (2015). A history (1990-2015) of mismanaging the vaquita into extinction - A Mexican NGO’s perspective. *Journal of Marine Animals and Their Ecololgy*, *8*, 15-25.

Ceron, C. A. A., De los Rios-Carmenado, I., & Fernández, S. M. (2018). Illicit crops substitution and rural prosperity in armed conflict areas: A conceptual proposal based on the Working with People model in Colombia. *Land Use Policy*, *72*, 201-214.

CIRVA (1997). Report of the First Meeting of the International Committee for the Recovery of the Vaquita (CIRVA) Ensenada, Baja California, México 25–26 January 1997. Available from <http://boycottmexicanshrimp.com/CIRVA-Reports-I-II-III-Vaquita-1997-1999-2004.pdf>

CIRVA (1999). Report of the Second Meeting of the International Committee for the Recovery of the Vaquita (CIRVA) Ensenada, Baja California, México 7-11 February 1999. Available from <http://boycottmexicanshrimp.com/CIRVA-Reports-I-II-III-Vaquita-1997-1999-2004.pdf>

CIRVA (2004). Report of the Third Meeting of the International Committee for the Recovery of the Vaquita (CIRVA) Ensenada, Baja California, México 18-24 January 2004. Available from <http://boycottmexicanshrimp.com/CIRVA-Reports-I-II-III-Vaquita-1997-1999-2004.pdf>

CIRVA (2012). Report of the Fourth Meeting of the International Committee for the Recovery of the Vaquita (CIRVA). Available from <https://www.iucn.org/sites/dev/files/import/downloads/report_of_the_fourth_meeting_of_the_international_committee_for_the_recovery_of_vaqui.pdf>

CIRVA (2014). Report of the Fifth Meeting of the International Committee for the Recovery of the Vaquita (CIRVA). Available from <http://www.iucn-csg.org/wp-content/uploads/2010/03/Report-of-the-Fifth-Meeting-of-CIRVA.pdf>

CIRVA (2015). Report of the Sixth Meeting of the International Committee for the Recovery of the Vaquita (CIRVA). Available from <http://iucn-csg.org/wp-content/uploads/2010/03/CIRVA-6-Report-Rev-19-July-2015.pdf>

CIRVA (2016). Report of the Seventh Meeting of the International Committee for the Recovery of the Vaquita (CIRVA). Available from <http://www.iucn-csg.org/wp-content/uploads/2010/03/CIRVA-7-Final-Report-June-23.pdf>

CIRVA (2017). Report of the Eighth Meeting of the International Committee for the Recovery of the Vaquita (CIRVA). Available from <http://www.iucn-csg.org/wp-content/uploads/2010/03/CIRVA-8-Report-Final.pdf>

CIRVA (2018). Report of the Ninth Meeting of the International Committee for the Recovery of the Vaquita (CIRVA). Available from <http://www.iucn-csg.org/wp-content/uploads/2010/03/CIRVA-9-Final-Report-May-11-2017.pdf>

CIRVA (2019). Report of the Ninth Meeting of the International Committee for the Recovery of the Vaquita (CIRVA). Available from <https://iucn-csg.org/wp-content/uploads/2018/01/CIRVA-10_final-report-2018.pdf>

Cope, D., Vickery, J., & Rowcliffe, M. (2005). From conflict to coexistence: a case study of geese and agriculture in Scotland. *Conservation Biology Series - Cambridge*, *9*, 176.

Crabtree, B., Humphreys, L., Moxey, M., & Wernham, C. (2010). Review of Goose Management Policy in Scotland. BTO Scotland report to Scottish Government. Available from [http://www.gov.scot/Resource/Doc/340628/0112833](http://www.gov.scot/Resource/Doc/340628/0112833.pdf)

Cubides, F., Mockus Sivickas, A., Avellaneda, M., González, H., Arcila Niño, Ó., Molano, A., ... & Mosquera Mesa, R. (1989). La Macarena: reserva biológica de la humanidad, territorio de conflicto.

D'agrosa, C., Lennert‐Cody, C. E., & Vidal, O. (2000). Vaquita bycatch in Mexico's artisanal gillnet fisheries: driving a small population to extinction. *Conservation Biology*, *14*(4), 1110-1119.

Dekker, L. (2018). A disagreement about livestock grazing in Enduimet Wildlife Management Area. MSc thesis, Wageningen University. Available at <https://edepot.wur.nl/442531>

Del Cairo, C., & Montenegro-Perini, I. (2015). Espacios, campesinos y subjetividades ambientales en el Guaviare. *Memoria y Sociedad*, *19*(39), 49-71.

DOF (1993). Norma Oficial Mexicana NOM-012-1993, por la que se establecen medidas para la protección de las especies de totoaba y vaquita en aguas de jurisdicción federal del Golfo de California. *Diario Oficial de la Federación*, Mexico.

DOF (1994). Norma Oficial Mexicana NOM-059-Ecol-1994, que determina las especies y subespecies de flora y fauna silvestres terrestres y acuáticas en peligro de extinción, amenazadas, raras y las sujetas a protección especial y que establece especificaciones para su protección. *Diario Oficial de la Federación, México.*

DOF (2005). “Programa de protección de la vaquita dentro de área de Refugio ubicada en la porción occidental del Alto Golfo de California”. In *Diario Oficial de la Federación, Septiembre del 2005*

European Commission (2007). Management Plan for Turtle Dove (*Streptopelia turtur*) 2007 – 2009. Available at <https://ec.europa.eu/environment/nature/conservation/wildbirds/hunting/docs/turtle_dove.pdf>

Flessa, K. W., Calderon-Aguilera, L., Cintra-Buenrostro, C. E., Dettman, D. L., Dietl, G. P., Goodwin, D. H., ... & Schöne, B. R. (2019). Vaquita face extinction from bycatch. Comment on Manjarrez-Bringas, N. et al., Lessons for sustainable development: Marine mammal conservation policies and its social and economic effects. Sustainability 2018, 10, 2185. *Sustainability*, *11*(7), 2161.

Gantiva, G. P., & Iregui, G. T. (1998). Políticas de desarrollo rural en el área de influencia del parque nacional natural sierra de la macarena. *Revista de la Facultad de Medicina Veterinaria y de Zootecnia*, *46*(2), 8-14.

Gerrodette, T., Taylor, B. L., Swift, R., Rankin, S., Jaramillo‐Legorreta, A. M., & Rojas‐Bracho, L. (2011). A combined visual and acoustic estimate of 2008 abundance, and change in abundance since 1997, for the vaquita, *Phocoena sinus*. *Marine Mammal Science*, *27*(2), E79-E100.

Homewood, K. M. (2017). “They Call It Shangri-La”: Sustainable Conservation, or African Enclosures?. In *The Anthropology of Sustainability* (pp. 91-109). Palgrave Macmillan, New York.

Honey, M. (2008) *Ecotourism and Sustainable Development. Who Owns Paradise?* Washington, DC: Island Press.

<https://era.ed.ac.uk/bitstream/handle/1842/36832/Baynham-Herd2020.pdf?sequence=1>

Jaramillo-Legorreta, A. M., Cardenas-Hinojosa, G., Nieto-Garcia, E., Rojas-Bracho, L., Thomas, L., Ver Hoef, J. M., ... & Tregenza, N. (2019). Decline towards extinction of Mexico's vaquita porpoise (*Phocoena sinus*). *Royal Society Open Science*, *6*(7), 190598. <https://doi.org/10.1098/rsos.190598>

Jaramillo‐Legorreta, A., Cardenas‐Hinojosa, G., Nieto‐Garcia, E., Rojas‐Bracho, L., Ver Hoef, J., Moore, J., ... & Taylor, B. (2017). Passive acoustic monitoring of the decline of Mexico's critically endangered vaquita. *Conservation Biology*, *31*(1), 183-191.

Kulindwa, K., Mvena, Z., & Runyoro, V. (2003). Baseline Study for the Proposed Pilot Wildlife Management Areas (WMAs). Ministry of Natural Resources and Tourism, Wildlife Division, Government of Tanzania. Available at <https://www.ucl.ac.uk/pima/docs/reference/05_main_baseline.pdf>

Mason, T. H., Keane, A., Redpath, S. M., & Bunnefeld, N. (2018). The changing environment of conservation conflict: geese and farming in Scotland. *Journal of Applied Ecology*, *55*(2), 651-662.

McKenzie, R. (2014). Islay Sustainable Goose Management Strategy. Scottish Natural Heritage. Available at <https://www.nature.scot/professional-advice/land-and-sea-management/managing-wildlife/managing-geese/islay-sustainable-goose-management-strategy>

McKenzie, R., & Shaw, J. M. (2017). Reconciling competing values placed upon goose populations: The evolution of and experiences from the Islay Sustainable Goose Management Strategy. *Ambio*, *46*(2), 198-209. <https://doi.org/10.1007/s13280-016-0880-8>

Mejía, D. (2016). Plan Colombia: An Analysis of Effectiveness and Costs. Available from <https://www.brookings.edu/wp-content/uploads/2016/07/Mejia-Colombia-final-2.pdf>

Minwary, M. Y. (2009). Politics of participatory wildlife management in Enduimet WMA, Tanzania. MSc thesis, Norwegian University of Life Sciences (UMB). Available at <http://citeseerx.ist.psu.edu/viewdoc/download?doi=10.1.1.468.8029&rep=rep1&type=pdf>

Nelson, F. (2004). *The evolution and impacts of community-based ecotourism in northern Tanzania* (No. 131). IIED, London.

Nelson, F. (2007). *Emerging or illusory? Community wildlife management in Tanzania*. IIED.

Nijhawan, S. (2018). *Human-animal relations and the role of cultural norms in tiger conservation in the Idu Mishmi of Arunachal Pradesh, India*. Doctoral dissertation, University College London. Available at <https://discovery.ucl.ac.uk/id/eprint/10041224/>

Nkwame, V. M. (2007). Villages granted total authority over wildlife. *Arusha Times*, (0856-9135), 18-24.

Ogilvie, M. A. (1992). Recent status change of some birds on Islay. *The Journal of the Scottish Ornithologists' Club*, *16*, 240-259.

Owen, M. (1977). The role of wildfowl refuges on agricultural land in lessening the conflict between farmers and geese in Britain. *Biological Conservation*, *11*(3), 209-222.

Owen, M. (1990). The damage‐conservation interface illustrated by geese. *Ibis*, *132*(2), 238-252.

Pennisi, E. (2017). After failed rescue effort, rare porpoise in extreme peril. *Science,* *358*, 851. 10.1126/science.358.6365.851

Percival, S. M. (1991). The population structure of Greenland Barnacle Geese *Branta leucopsis* on the wintering grounds on Islay. *Ibis*, *133*(4), 357-364.

Percival, S. M., Halpin, Y., & Houston, D. C. (1997). Managing the distribution of barnacle geese on Islay, Scotland, through deliberate human disturbance. *Biological Conservation*, *82*(3), 273-277.

Puentes Casas, E. (2013). Políticas ambientales de conservación y conflictos en áreas protegidas: El caso del Parque Nacional natural “Sierra de la Macarena”, Colombia (1948-2009). Available from <http://repositorio.filo.uba.ar/bitstream/handle/filodigital/1841/uba_ffyl_t_2013_se_puentes.pdf?sequence=1&isAllowed=y>

Quintero, G. J. T., & Restrepo, G. I. (2009). Erradicación de cultivos ilícitos y desplazamiento forzado en el parque natural Sierra de la Macarena. *Cuadernos de Desarrollo Rural*, *6*(63), 107-138.

Reardon, S. (2018). FARC and the forest: Peace is destroying Colombia's jungle--and opening it to science. *Nature*, *558*(7709), 169-169.

Rojas-Bracho, L., & Fueyo, L. (2010). Helping the vaquita to recover—conservation and management actions by Mexico. *Journal of the American Cetacean Society*, *39*, 7-10.

Rojas-Bracho, L., & Reeves, R. (2013). Vaquitas and gillnets: Mexico’s ultimate cetacean conservation challenge. *Endangered Species Research,* *21*, 77–87. <https://doi.org/10.3354/esr00501>

Rojas-Bracho, L., Gulland, F. M. D., Smith, C. R., Taylor, B., Wells, R. S., Thomas, P. O., ... & Balle, J. D. (2019). A field effort to capture critically endangered vaquitas Phocoena sinus for protection from entanglement in illegal gillnets. *Endangered Species Research*, *38*, 11-27.

Rojas‐Bracho, L., Reeves, R. R., & Jaramillo‐Legorreta, A. (2006). Conservation of the vaquita *Phocoena sinus*. *Mammal Review*, *36*(3), 179-216.

Sandoval, P. J. M., Van Dexter, K., Van Den Hoek, J., Wrathall, D., & Kennedy, R. E. (2020). The end of gunpoint conservation: Forest disturbance after the Colombian peace agreement. *Environmental Research Letters*, *15*, 030433. <https://doi.org/10.1088/1748-9326/ab6ae3>

SEMARNAT (2008). Programa de Acción para la Conservación de la Especie: Vaquita (*Phocoena sinus*). Estrategia integral para el manejo sustentable de los recursos marinos y costeros en el Alto Golfo de California. Secretaría de Medio Ambiente y Recursos Naturales. 73 pp. Available from <http://www.conanp.gob.mx/pdf_especies/PACEvaquita.pdf>

Serna, D. R. (2003). Campesinos entre la selva, invasores de reservas. *Tabula Rasa*, (1), 183-210.

Sulle, E., Lekaita, E., & Nelson, F. (2011). From Promise to Performance? wildlife management areas in Northern Tanzania. In *Tanzania Natural Resource Forum and Maliasili Initiatives*. Available at <https://www.ucl.ac.uk/pima/docs/reference/13_promise_to_performance.pdf>

Taylor, B. L., Rojas‐Bracho, L., Moore, J., Jaramillo‐Legorreta, A., Ver Hoef, J. M., Cardenas‐Hinojosa, G., ... & Thomas, L. (2017). Extinction is imminent for Mexico's endemic porpoise unless fishery bycatch is eliminated. *Conservation Letters*, *10*(5), 588-595.

Trench, P. C., Kiruswa, S., Nelson, F., & Homewood, K. (2009). Still “People of Cattle”? Livelihoods, diversification and community conservation in Longido District. In *Staying Maasai?* (pp. 217-256). New York, NY: Springer.

UAESPNN-Unidad Administrativa Especial del Sistema de Parques Nacionales Naturales (2005) Plan de Manejo Básico Parque Nacional Natural Sierra de la Macarena 2005-2009 (versión 2009).

United Republic of Tanzania (2007). *Wildlife Policy*. Dar es Salaam, United Republic of Tanzania.

Villa-Ramírez, B. (1993). Recovery plan for the vaquita, *Phocoena sinus*. *Departamento de Zoología, Instituto de Biología, Universidad Nacional Autónoma de México, México, DF*.

Whitehouse, A. (2009). A Disgrace to a Farmer’ Conservation and Agriculture on a Nature Reserve in Islay, Scotland. *Conservation and Society*, *7*(3), 165-175.

Wright, V. C. (2016). Turbulent times: fighting history today in Tanzania’s trophy hunting spaces. *Journal of Contemporary African Studies*, *34*(1), 40-60.

Wright, V. C. (2017). Turbulent terrains: The contradictions and politics of decentralised conservation. *Conservation and Society*, *15*(2), 157-167.
