# Supplementary material for: Measuring the intensity of conflicts in conservation
Source: Conserv Lett. 2021 Jan 11;14(3):e12783. doi: 10.1111/conl.12783 (PMC8365684; doi:10.1111/conl.12783)
Supplement: Supplementary file 3 — Supplementary Material [file CONL-14-e12783-s001.docx]

**Supporting Information S3**: Qualitative analysis of conflict phases across the seven case studies.

**Conflict escalation**

Of the seven case studies, three demonstrated a clear initial sequence of conflict escalation following the trigger. This escalation generally started at conflict intensity levels of 1 (latent conflict) or 2 (expressed disagreement) and progressed through to level 4 over time. For example, following the establishment of Dibang Valley Wildlife Sanctuary (DWLS) in the Indian state of Arunachal Pradesh without the consent of local people, a prolonged period of latent conflict was observed during which the sanctuary largely functioned as a paper park with little enforcement and minimal impact on local resource use (Nijhawan, 2018). Cultural acceptance allowed endangered wildlife to coexist with people, despite occasional instances of negative human-wildlife interactions. The situation changed in 2012 when two tiger cubs were rescued from a well on the outskirts of DWLS, which prompted renewed interest in upgrading the sanctuary as a strict tiger reserve, thereby changing the relationship with local people who felt abandoned in favour of wildlife (Aiyadurai, 2016). The situation in Dibang Valley contrasts with that of the Macarena Conservation Area, Colombia, in which poor delineation and enforcement of protected area boundaries resulted in rapid settlement by local people displaced by sustained political and military unrest (UNDP, 2014).

**Conflict stasis and surge**

For most case studies, conflict intensity was found to peak and oscillate between levels 3 and 4, often with periods of stasis at either of these levels. For example, the conflict between European turtle dove (*Streptopelia turtur*) conservation and hunting in Spain has mostly remained at level 4 since 2015, with conservation groups and hunting associations respectively promoting and resisting attempts at implementing a hunting moratorium (Lormée et al., 2019). A lull in the intensity of the conflict did occur in 2017 as a delayed response to the development of an action plan for the management of the species the previous year. Stasis was also observed to occur at lower levels of conflict, and particularly as a result of successful mitigation policy. For example, the implementation of consecutive management policies involving compensation payments in 1988 and 1992, and both compensation and culling in 2000, by Scottish National Heritage to mitigate the conflict between goose conservation and farming interests on Islay, Scotland, led to prolonged periods of level 2 conflict. These periods of stasis contrast with rapid peaks at level 4, reflecting surges in conflict brought about by a rapid succession of stakeholder actions. For example, in the case of the management of baboons in urban areas of Cape Peninsula, South Africa, built up disagreement between stakeholders involved in the Baboon Management Team (BMT) led to a formal complaint by scientists and the restructuring of the BMT in 2002 (Koutstall, 2013). These changes were preceded and followed by periods of level 2 conflict during which stakeholders expressed disagreement about baboon management.

**Violent conflict**

Of the seven case studies, two involved instances of physical violence between stakeholder groups. As an example, with a global population of only 19 individuals, efforts to protect the critically endangered vaquita (*Phocoena sinus*) from unintended by-catch in gillnets have resulted in a consistently intense conflict between the local fishing communities and conservation efforts by NGOs and the Mexican government (Jaramillo-Legorreta et al., 2019). Following the designation of protected areas for the species and a ban on gillnet fishing, military enforcement was brought in to curb illegal gillnet fishing, leading to occasional violent clashes with fishers.

**Conflict de-escalation**

It is notable that no case study exhibited long-lasting de-escalation of conflict intensity. Rather, temporary reductions in conflict intensity often occurred following rapid peaks at level 4, reflecting intense efforts by multiple stakeholders to address a given conflict. For instance, a combination of collaborative actions – including anti-poaching efforts and elephant crop-foraging mitigation – carried out by local authorities and villages in Enduimet Wildlife Mangement Area in Tanzania successfully brought the conflict down to level 0 in 2013 (Homewood, 2017).

**References**

Aiyadurai, A. (2016). ‘Tigers are Our Brothers’ Understanding Human-Nature Relations in the Mishmi Hills, Northeast India. *Conservation and Society*, *14*(4), 305-316.

Jaramillo-Legorreta, A. M., Cardenas-Hinojosa, G., Nieto-Garcia, E., Rojas-Bracho, L., Thomas, L., Ver Hoef, J. M., ... & Tregenza, N. (2019). Decline towards extinction of Mexico's vaquita porpoise (Phocoena sinus). *Royal Society Open Science*, *6*(7), 190598. <https://doi.org/10.1098/rsos.190598>

Kansky, R., Kidd, M., & Knight, A. T. (2016). A wildlife tolerance model and case study for understanding human wildlife conflicts. *Biological Conservation*, *201*, 137-145. <https://doi.org/10.1016/j.biocon.2016.07.002>

Lormée, H., Barbraud, C., Peach, W., Carboneras, C., Lebreton, J. D., Moreno-Zarate, L. A. R. A., ... & Eraud, C. (2019). Assessing the sustainability of harvest of the European Turtle-dove along the European western flyway. *Bird Conservation International*, 1-16.  <https://doi.org/10.1017/S0959270919000479>

McKenzie, R., & Shaw, J. M. (2017). Reconciling competing values placed upon goose populations: The evolution of and experiences from the Islay Sustainable Goose Management Strategy. *Ambio*, *46*(2), 198-209. <https://doi.org/10.1007/s13280-016-0880-8>

Nijhawan, S. (2018). *Human-animal relations and the role of cultural norms in tiger conservation in the Idu Mishmi of Arunachal Pradesh, India*. Doctoral dissertation, University College London.

Trench, P. C., Kiruswa, S., Nelson, F., & Homewood, K. (2009). Still “People of Cattle”? Livelihoods, diversification and community conservation in Longido District. In *Staying Maasai?* (pp. 217-256). New York, NY: Springer.

United Nations Development Programme. (2014). *Human Development Report 2014: Sustaining Human Progress: Reducing Vulnerabilty and Builing Resilience*. New York, NY: UNDP.

Homewood, K. M. (2017). “They Call It Shangri-La”: Sustainable Conservation, or African Enclosures?. In *The Anthropology of Sustainability* (pp. 91-109). Palgrave Macmillan, New York.
