# Supplementary material for: Measuring the intensity of conflicts in conservation
Source: Conserv Lett. 2021 Jan 11;14(3):e12783. doi: 10.1111/conl.12783 (PMC8365684; doi:10.1111/conl.12783)
Supplement: Supplementary file 4 — Supplementary Material [file CONL-14-e12783-s002.docx]

**Supporting Information S4**: Results of mixed effects ordinal regression models.

**Table S4.1** – AIC-based model comparison results for mixed effects ordinal regression models. All models included case study as a random intercept. The shaded row denotes the best model.

| Model structure | # parameters | Log-likelihood | AIC |
| --- | --- | --- | --- |
| ~ Collab + YST | 6 | -150.4 | 312.7 |
| ~ Collab | 5 | -153.1 | 316.1 |
| ~ YST | 5 | -154.3 | 318.6 |
| ~ 1 | 4 | -159.1 | 326.1 |

**Figure S4.1** - Effects of proportion of collaborative actions at time *t-1* (a) and number of years since the conflict trigger (b) on the probability of observing conflict levels 1-4. Relationships were obtained from a mixed effects ordinal regression model that included both variables as additive fixed effects and case study as a random intercept. Full and dashed lines denote predicted probabilities and 95% confidence intervals, respectively.
